# Anti-inflammatory Activity of a Polypeptide Fraction From *Achyranthes bidentate* in Amyloid β Oligomers Induced Model of Alzheimer’s Disease

**DOI:** 10.3389/fphar.2021.716177

**Published:** 2021-08-12

**Authors:** Xiangyu Ge, Yitong Wang, Shu Yu, Xuemin Cao, Yicong Chen, Qiong Cheng, Fei Ding

**Affiliations:** ^1^ Key Laboratory of Neuroregeneration of Jiangsu and Ministry of Education, Jiangsu Province Co-innovation Center of Neuroregeneration, NMPA Key Laboratory for Research and Evaluation of Tissue Engineering Technology Products, Nantong University, Nantong, China; ^2^ School of Medicine, Nantong University, Nantong, China; ^3^ Jiangsu Clinical Medicine Center of Tissue Engineering and Nerve Injury Repair, Nantong, China

**Keywords:** Achyranthes bidentate polypeptides, Aβ oligomers, microglia, polarization, neuroinflammation, neurotoxicity

## Abstract

Neuroinflammation plays a crucial role in neurodegenerative diseases such as Alzheimer’s disease (AD) and Parkinson’s disease (PD), and anti-inflammation has been considered as a potential therapeutic strategy. *Achyranthes bidentate* polypeptide fraction k (ABPPk) was shown to protect neurons from death and suppress microglia and astrocyte activation in PD model mice. However, how ABPPk regulates neuroinflammation to exert a neuroprotective role remains unclear. Toxic Aβ oligomers (AβOs) can trigger inflammatory response and play an important role in the pathogenesis of AD. In the present study, for the first time, we investigated the effects and underlying mechanisms of ABPPk on neuroinflammation in AβOs-induced models of AD. *In vitro*, ABPPk pretreatment dose-dependently inhibited AβOs-induced pro-inflammatory cytokines mRNA levels in BV2 and primary microglia. ABPPk pretreatment also reduced the neurotoxicity of BV2 microglia-conditioned media on primary hippocampal neurons. Furthermore, ABPPk down-regulated the AβOs-induced phosphorylation of IκBα and NF-κB p65 as well as the expression of NLRP3 in BV2 microglia. *In vivo*, ABPPk pre-administration significantly improved locomotor activity, alleviated memory deficits, and rescued neuronal degeneration and loss in the hippocampus of AβOs-injected mice. ABPPk inhibited the activation of microglia in hippocampal CA3 region and suppressed the activation of NF-κB as well as the expression of NLRP3, cleaved caspase-1, and ASC in the brain after AβOs injection. ABPPk hindered the release of pro-inflammatory cytokines and promoted the release of anti-inflammatory cytokines in the brain. Notably, the polarization experiment on BV2 microglia demonstrated that ABPPk inhibited M1-phenotype polarization and promoted M2-phenotype polarization by activating the LPS- or AβOs-impaired autophagy in microglia. Taken together, our observations indicate that ABPPk can restore the autophagy of microglia damaged by AβOs, thereby promoting M2-phenotype polarization and inhibiting M1-phenotype polarization, thus playing a role in regulating neuroinflammation and alleviating neurotoxicity.

## Introduction

Alzheimer’s disease (AD) is a progressive neurodegenerative disorder characterized by amyloid-beta (Aβ) plaques, neurofibrillary tangles (NFTs), and neuroinflammation (Probst et al., 1991; Webers et al., 2020). As a hallmark of neuroinflammation, microglia activation has received increasing attention in the pathogenesis of AD. Excessive production and deposition of Aβ has been considered as the principal inducer of microglia activation and neuroinflammation in the AD brain (Selkoe, 2001). Recent genome-wide association studies have also shown that most of the AD risk loci can be found in genes highly or uniquely expressed in microglia (Verheijen and Sleegers, 2018). Compelling evidence has indicated the dual roles of microglia in the pathogenesis of AD. On the one hand, microglia can play a beneficial role in the pathogenesis of AD by producing anti-inflammatory mediators and clearing amyloid plaque (Lyman et al., 2014; Morales et al., 2014). On the other hand, activated microglia and released inflammatory mediators can enhance Aβ deposition and aggravate neuroinflammation, induce the formation of a vicious cycle, and eventually lead to irreversible loss of neurons (Cai et al., 2014).

With the development of research on neuroinflammation, anti-inflammatory therapy has been considered as a potential treatment for AD. However, a comparative analysis showed that traditional non-steroidal anti-inflammatory drugs, aspirin and steroids as well as selective COX-2 inhibitors could not significantly improve the cognitive decline. Instead, these treatments might cause bleeding, hyperglycemia, nausea, hypertension, and other side effects (Jaturapatporn et al., 2012). Numerous studies have shown that natural products such as terpenoids, saponins, alkaloids, flavonoids, polyphenols, and coumarins have multifunctional properties and are capable of interacting concurrently with multiple targets of neuroinflammation in AD (Ahmed et al., 2017; Shal et al., 2018; Olajide and Sarker, 2020; Patil et al., 2020). Thus, there are growing attempts to develop drugs from natural products to treat neurodegenerative diseases.

*Achyranthes bidentate* (*A. bidentate*) has been commonly used in the treatment of dementia and osteoporosis in the traditional Chinse medicine for thousands of years. The Chinese, Japanese, and Korean Pharmacopeias have all documented the wide range of pharmacological effects of the dried roots of *A. bidentate*. In the immune and nervous systems, *A. bidentate* possesses anti-inflammatory and antioxidant biological activities. Phytochemical investigations revealed that the major bioactive substances of *A. bidentate* are polysaccharides, polypeptides, triterpenoid saponins, and ketosteroids (He et al., 2017). Studies have shown that steroid-enriched fraction of *A. bidentate* can protect Aβ-induced cognitive impairment and suppress neuroinflammation in rats (Lin et al., 2019). Geniposide, an iridoid glycoside compound contained in *A. bidentate*, has neuroprotective, antioxidant, anti-inflammatory, anti-depressive, and other benefiting effects, and may be used to prevent and treat AD (Zhou et al., 2019). Previous study has shown that *A. bidentate* polypeptide fraction k (ABPPk) can attenuate microglia activation and down-regulate neuroinflammation in 1-methyl-4-pheynl-1,2,3,6-tetrahydropyridine hydrochloride (MPTP)-induced Parkinson’s model mice (Peng et al., 2018). Recently, *in vitro* experiments also showed that ABPPk can inhibit the release of TNF-α, IL-1β, NO, and PEG_2_ from LPS-stimulated BV2 microglia by inhibiting the activation of nuclear factor κB (NF-κB) and promoting the expression and translocation of nuclear factor erythroid 2-related factor 2 (Nrf2) (Cheng et al., 2019). NF-κB can bind to the promoter regions of many genes involved in amyloidogenesis and inflammation, and is considered as a reasonable target against AD pathology (Ju Hwang et al., 2019; Thawkar and Kaur, 2019). Nrf2 protein is involved in detoxification, repair and clearance of damaged proteins and organelles, inflammation, mitochondrial function, and antioxidant processes (Dinkova-Kostova et al., 2018). In AD patients, the level of Nrf2 in the nucleus is reduced (Rojo et al., 2017). Nrf2 activation may reduce oxidative stress and chronic inflammation in human AD (Prasad, 2016). We speculate that ABPPk may play a positive role in the regulation of AD neuroinflammation. Therefore, in this study, we used Aβ oligomers (AβOs)-induced *in vitro* and *in vivo* models of AD to investigate the effects and the underlying mechanism of ABPPk on AβOs-induced neuroinflammation and neurotoxicity.

## Materials and Methods

### Extraction, Isolation and Identification of *A. bidentate* Polypeptide Fraction k

Roots of *A. bidentate* were purchased from a local Chinese medicine grocer and identified by the pharmacist. The powders of *A. bidentate* polypeptide fraction k (ABPPk) were extracted according to the previous studies (Yuan et al., 2010; Cheng et al., 2014). Dried ABPPk powders were easily dissolved in PBS to achieve a desired concentration. The used concentration of ABPPk (2.5, 5, and 10 μg/ml) *in vitro* and the dose of ABPPk (2.5 mg/kg) *in vivo* were based on the previous studies (Cheng et al., 2018; Cheng et al., 2019).

### Aβ Oligomers Preparation

AβOs preparation was performed as previously described with some modification (Dahlgren et al., 2002). Briefly, 1 mg Aβ protein fragment 1-42 (No. A9810, Merck, United States) was dissolved in 1 ml pre-cooled 1,1,1,3,3,3, -Hexafluoro-2-propanol (HFIP, No. H107503, Aladdin, China). After removing HFIP under vacuum by a refrigerated centrifuge concentrator, the peptide film was dissolved in 2 ml DMSO (No. D8418, Merck, United States) to obtain a 1 mM solution then further diluted in PBS (pH 7.4) to make a 100 μM stock solution. The stock solution was incubated at 4°C for 24 h to prepare the Aβ oligomers (AβOs). AβOs stock solution was stored at −80°C and prepared to the designed concentration for the experiment before use. The concentrations used for AβOs *in vitro* (10 μM) and *in vivo* (80 μM) referred to the reported literatures (Song et al., 2017; Batista et al., 2021).

### Cell Culture and Treatment

BV2 microglia were purchased from the Institute of Basic Medical Sciences of the China Science Academy and were cultured in Dulbecco’s Modified Eagle Medium (DMEM) supplemented with 10% fetal bovine serum (FBS) in a 5% CO_2_ incubator at 37°C. When cells need to be passaged, they were digested with 0.25% trypsin-EDTA and then planted in different culture plates or dishes according to the required density (2 × 10^5^ cells/ml for immunocytochemistry, 1 × 10^6^ cells/ml for qPCR and Western blot). The cells were pre-treated with different concentration of ABPPk (2.5 μg/ml, 5 μg/ml, and 10 μg/ml) for 30 min and then cultured with 10 μM AβOs for 24 h for subsequent experiments. Primary microglia were isolated from the brain of newborn 1 day C57BL/6 mouse pups with some modifications as described previously (Sepulveda-Diaz et al., 2016). Briefly, the cortical and hippocampal tissues were digested with 0.125% trypsin at 37°C for 15 min. After termination of digestion, the homogeneous cell suspension was plated in poly-L-lysine (PLL) coated Corning T-75 flasks containing DMEM supplemented with 10% FBS and antibiotics. The culture medium was changed the next day to remove cell debris, and then changed on the 5th day. On the 8th day of culture, the top layer of microglial cells was collected gently by using 0.05% trypsin and re-seeded in PLL-coated culture plates at a proper density (1 × 10^5^ cells/ml for immunocytochemistry, 1 × 10^6^ cells/ml for qPCR). For qPCR, primary microglia were pre-treated with different concentrations of ABPPk (2.5 μg/ml, 5 μg/ml, and 10 μg/ml) followed by stimulation with 10 μM AβOs for 24 h.

Primary culture of hippocampal neurons were obtained from postnatal day 1 mouse pups as previously described (Moutin et al., 2020). In brief, hippocampi were digested with papain at 37°C for 10 min then followed by an additional 5 min in presence of DNase1. Complete medium (Neurobasal-A containing 2% B27, 0.25% GlutaMAX, 0.25% L-glutamine and 10% FBS) was added to terminate papain digestion. The tissues were mechanically dissociated with 1000 μl tips and 4% BSA buffer was added to make cells suspension. Then the cells were centrifuged at 1200 rpm for 7 min, re-suspended in complete medium and seeded on PLL and laminin pre-coated 96-well culture plates at the density of 1 × 10^6^ cells/ml. After incubated for 4 h, the medium was replaced by serum-free BrainPhys containing 2% B27 supplement, 0.25% GlutaMAX, and 1% antibiotics for further incubation. 8 h later, 5 μM cytosine β-D-arabinofuranoside hydrochloride (Ara-C, No. 1162002, Merck, United States) was added in the medium to inhibit glia proliferation. Afterwards, 80% of the medium was replaced by fresh one, twice a week. The neurons were cultured for 7 days for subsequent experiments.

### Animals and Aβ Oligomers Intracerebroventricular Injection

All the experiments were carried out in accordance with the Guide for the Care and Use of Laboratory Animals of the National Institutes of Health and approved by the Animal Experiments Ethical Committee of Nantong University. A total of 45 male C57BL/6 mice (28 ± 2 g, 8 weeks old) were randomly separated into three groups: sham operation (Sham group), AβOs (80 μM) injection (AβOs group), and ABPPk (2.5 mg/kg) pre-administration followed by AβOs (80 μM) injection (ABPPk group). Mice were anesthetized by intraperitoneal injection of avertin (1.25% bromethol, 0.2 ml/10 g body weight) (EasyCheck, AIBI Bio-Tech, Nanjing, China). A volume of 5 μl of AβOs (80 μM) was injected using a microsyringe which was inserted perpendicularly through the skull according to the following coordinates: anteroposterior (AP) −0.9 mm, mediolateral (ML) +1.7 mm, dorsoventral (DV) −2.2 mm from bregma (Kim et al., 2016). The injection was finished within 5 min, then the needle was retained for 2 min and withdrawn slowly. In the sham group, only the skull was exposed, but no injections were given. In the ABPPk group, 5 μl of ABPPk (2.5 mg/kg) was administered intracerebroventricularly 15 min prior to AβOs injection. The AβOs group was given equal volume of PBS. The body temperature of mice was maintained at 37°C during surgery. Intraperitoneal injection of 0.03 mg/kg buprenorphine was used for postop analgesia 2 h after surgery.

### BV2 Microglia-Conditioned Media System

BV2 microglia-CM system was prepared as previously described (Urrutia et al., 2017). BV2 microglia were seeded at a density of 2 × 10^5^ cells/ml in 6-well culture plates. After the cells were pre-treated with 5 μg/ml or 10 μg/ml ABPPk for 30 min, 10 μM AβOs were added in the culture media. After 24 h, the culture media were collected and centrifuged at 1200 rpm for 5 min to remove the suspended cells and obtain the supernatant. The supernatant was mixed with fresh complete BrainPhys culture medium at a 1:1 volume ratio to stimulate hippocampal neurons. The neuronal viability was measured using a cell counting kit (CCK-8 kit, Dojindo, Japan). Quantitative analysis of cytotoxicity was achieved by detecting the activity of lactate dehydrogenase (LDH) released into the culture medium from a ruptured plasma membrane cell with the LDH cytotoxicity assay kit (No. C0017, Beyotime, China).

### Quantitative Real Time PCR

qPCR was used to detect the effect of ABPPk on AβOs-induced changes of inflammatory cytokines mRNA levels in BV2 microglia. Total RNA from BV2 microglia was DNase-treated and purified by using the RNeasy Mini Kit (QIAGEN, Valencia, CA) according to the manufacturer’s instructions. Then cDNA was synthesized from the total RNA using Omniscript RT kit (QIAGEN) as per the manufacturer’s instructions. qPCR was performed using SYBR Green Supermix (Bio-Rad, Hercules, CA) and the Stepone RT-PCR instrument (Applied Biosystems, Foster City, CA). The housekeeping gene Gapdh was used as an internal control. Primers were designed and synthesized by GENEray biotechnology (Shanghai, China). The primers used in this study were listed in Table 1.

**TABLE 1:** Primers used for qPCR.

Transcript	Forward Primer	Reverse Primer
Mouse Arg1	TTG​GGT​GGA​TGC​TCA​CAC​TG	GTA​CAC​GAT​GTC​TTT​GGC​AGA
Mouse CD206	CTC​TGT​TCA​GCT​ATT​GGA​CGC	TGG​CAC​TCC​CAA​ACA​TAA​TTT​GA
Mouse IL-1β	GAG​AGC​ATC​CAG​CTT​CAA​A	TCATCATCCCACGAGTCA
Mouse IL-4	GGCAACAAGGAACACCAC	CACCGAGAACCCCAGAC
Mouse IL-6	CAC​CAG​GAA​CGA​AAG​TCA​A	CAA​CAA​CAT​CAG​TCC​CAA​GA
Mouse IL-10	AGGGTTACTTGGGTTGCC	GGG​TCT​TCA​GCT​TCT​CTC​C
Mouse IL-13	CAGCATGGTATGGAGCGT	CTGGGTCCTGTGGATGG
Mouse IL-18	AAC​GAA​TCC​CAG​ACC​AGA​C	AGA​GGG​TAG​ACA​TCC​TTC​CAT
Mouse TGF-β	GCA​GGA​AGA​GAA​GCC​AGC​A	GACAGCCAGGGCCACAA
Mouse TNF-α	CCACCACGCTCTTCTGTC	GCTACGGGCTTGTCACTC
Mouse Gapdh	CGT​ATT​GGG​CGC​CTG​GTC​ACC​AG	GAC​CTT​GCC​CAC​AGC​CTT​GGC​AGC

### Immunocytochemistry

Following treatments, primary microglia or BV2 microglia on coverslips were fixed with 4% paraformaldehyde (PFA) for 20 min at room temperature and later washed with PBS. After permeabilization and blocking with PBS containing 5% BSA and 0.1% Triton X-100, coverslips were incubated at 4°C overnight with the primary antibody Iba-1 (Abcam, ab178846, 1:500), CD86 (a marker for M1-type microglia, Abcam, ab220188, 1:50), CD206 (a marker for M2-type microglia, Abcam, ab64693, 1:200), phosphor-NF-κB p65 (Ser536) (p-p65, Abcam, ab16502, 1:200), and LC3B (a marker for autophagic flux, Cell signaling technology, 83506S, 1:400), then with the secondary antibody for 2 h at room temperature after washing with PBS. Then coverslips were mounted on microscope slides with mounting medium containing DAPI (Vector labs, United States) for photographing under Zeiss Axio Imager M2 fluorescence microscope (Zeiss, Germany). All *in vitro* experiments were repeated independently three times. For the quantitative analysis, two different slides of each group were investigated by a blinder and the mean fluorescence intensity of CD86 or CD206 was analyzed by ImageJ (NIH Image, Washington, United States).

### Open Field Test

Mice were allowed to acclimate the open field test apparatus for 3 days before stereotactic injection. Open field test was conducted at 24 h after AβOs injection. Each mouse was placed in a 50–50 cm square open box divided into a perimeter and a central area with 35-cm-high walls around. To start the experiment, the mice were gently placed in a corner of the box and a video camera was used to observe the total distance the mice traveled, the time they spent in the inner area and the number of times they stood on their hind limbs over a period of 5 minutes. ANY-maze behavioral tracking software (Stoelting Co., United States) was used to record and analyze locomotor activity of each mouse (*n* = 15 mice per group).

### Morris Water Maze Test

Morris water maze test was performed as previously described (Vorhees and Williams, 2006). Briefly, mice were trained four times a day for 4 days before stereotactic injection. During training, a platform was placed in a fixed position in the target quadrant 1 cm below the surface of the water. The mice were placed from different quadrants into the water for 90 s at a time, until they reached the platform. Mice that cannot find the platform within 90 s will be guided to the platform and allowed to remain there for 10 s. 24 h after the AβOs injection, the platform was removed entirely. Mouse was placed into the water for 90 s. The latency for each mouse (*n* = 15 mice per group) to enter the target quadrant, the frequency to enter the platform area, the total path and swimming time in the target quadrant were recorded by using ANY-maze Video Tracking System (Steolting Co.).

### Fluoro-Jade C Staining

After behavior test, three mice of each group were anesthetized with isoflurane and transcardially perfused with saline via the left ventricle, followed by 4% PFA for histology. The brain was removed and stored overnight in 4% PFA at 4°C, followed by gradient dehydration with sucrose, embedding with OCT, and continuous freezing of coronal sections (20-μm thick) in bregma −1.2 to −2.2 mm for Fluoro-Jade C (FJC) staining to identify neuronal cell death. The slices were incubated in 0.06% potassium permanganate for 10 min, and then immersed in FJC (No. AG325, Chemicon, United States) staining solution for 30 min. Two different sections of each mouse were photographed on the fluorescence microscope. FJC-positive cells in the hippocampus were counted by a blinded investigator with the Image J software. Data are presented as the mean of total FJC-positive cells for each mouse.

### Immunohistochemistry

Frozen brain sections (8-μm thick) were used for immunohistochemical analysis. The primary antibodies were neuronal nuclei (NeuN, a marker for neurons, Abcam, ab104224, 1:600), Iba-1 (a marker for microglia, Wako, 019-19741, 1:300), and p-p65 (Abcam, ab16502, 1:200). Isotype-matched antibodies were used as negative controls. After primary antibody incubation at 4°C, sections were incubated with Alexa fluorochrome-conjugated secondary antibodies (Invitrogen, 1:1000) for 1 h at room temperature. Brain sections were directly coverslipped with VECTASHIELD mounting medium containing DAPI (Vector labs, United States). Three different sections of each mouse were photographed on the fluorescence microscope (*n* = 3 mice per group). NeuN-positive cells, and Iba-1-positive cells were counted by a blinded investigator. Data are presented as the density of immunoreactive cells relative to the imaged area (mm^2^).

### Enzyme-Linked Immunosorbent Assay

After sacrificed, the mouse brain was removed quickly. Brain tissues from each group (*n* = 3 mice per group) were washed with pre-cooled saline, dried with filter paper and weighed. Pre-cooled homogenization medium (0.01 M Tris-HCl, 0.0001 M EDTA-2Na, 0.01 mol/l glucose, 0.8% NaCl, pH 7.4) was added proportionally (0.01 ml/mg), homogenated in ice bath for 3 min, then centrifuged 15,000 rpm at 4°C for 10 min. The supernatant was collected. Series of ELISA kits (IBL international, Hamburg, Germany) were used to detect the contents of IL-1β, IL-18, IL-4, IL-10, and TGF-β in the supernatant according to the manufacturer’s instructions. Absorbance was measured at 450 nm using a microplate reader (BioTek, Vermont, United States). The BCA kit was used to quantify the total protein content in the supernatant. The content of cytokines was calculated as per gram of protein (pg/g).

### Western Blot

The whole cell lysates of BV2 microglia or brain tissue samples (*n* = 3 mice per group) were extracted and collected by centrifugation (13,000 rpm, 10 min, 4°C). Protein concentrations were determined with Pierce™ BCA protein assay. Western blot analysis was performed as standard protocol. Protein samples were separated on 4–15% Tris-Gly SDS-PAGE gels (Beyotime, Shanghai, China) then transferred to PVDF membranes (Millipore, Bedford, United States). After blocking with TBST (5% non-fat dry milk or 5% BSA), the membranes were incubated with the primary antibody for phospho-IκBα (Ser32/36) (Cell signaling technology, 9246S, 1:1000), phospho-p65 (Abcam, ab16502, 0.5 μg/ml), NLRP3 (Cell signaling technology, 15101S, 1:1000), caspase-1 (Abcam, ab207802, 1:1000), TMS1/ASC (Abcam, ab151700, 1:5000), LC3B (Cell signaling technology, 83506S, 1:1000), SQSTM1/p62 (Abcam, ab109012, 1:10000), and Beclin1 (Abcam, ab62557, 1 μg/ml) at 4°C overnight. The membranes were incubated with HRP-conjugated secondary antibody for 1 h at room temperature. After thoroughly washed by TBST, membranes were incubated with the ECL western blotting substrate to detect the proteins. β-actin (Cell signaling technology, 3700S, 1:1000) served as loading control. ImageJ software was used to quantify the protein blots.

### Statistical Analysis

All data were presented as mean ± SD using Graph Pad Prism 8.0 (Graph Pad software, San Diego, United States). The unpaired two-tailed *t*-test was applied for comparisons between two groups. One-way ANOVA analysis was applied followed by Bonferroni post hoc testing for multiple comparisons. A *p*-value less than 0.05 was considered statistically significant.

## Results

### Chromatographic and Mass Spectrometry Analysis of *A. bidentate* Polypeptide Fraction k

**FIGURE 1:**
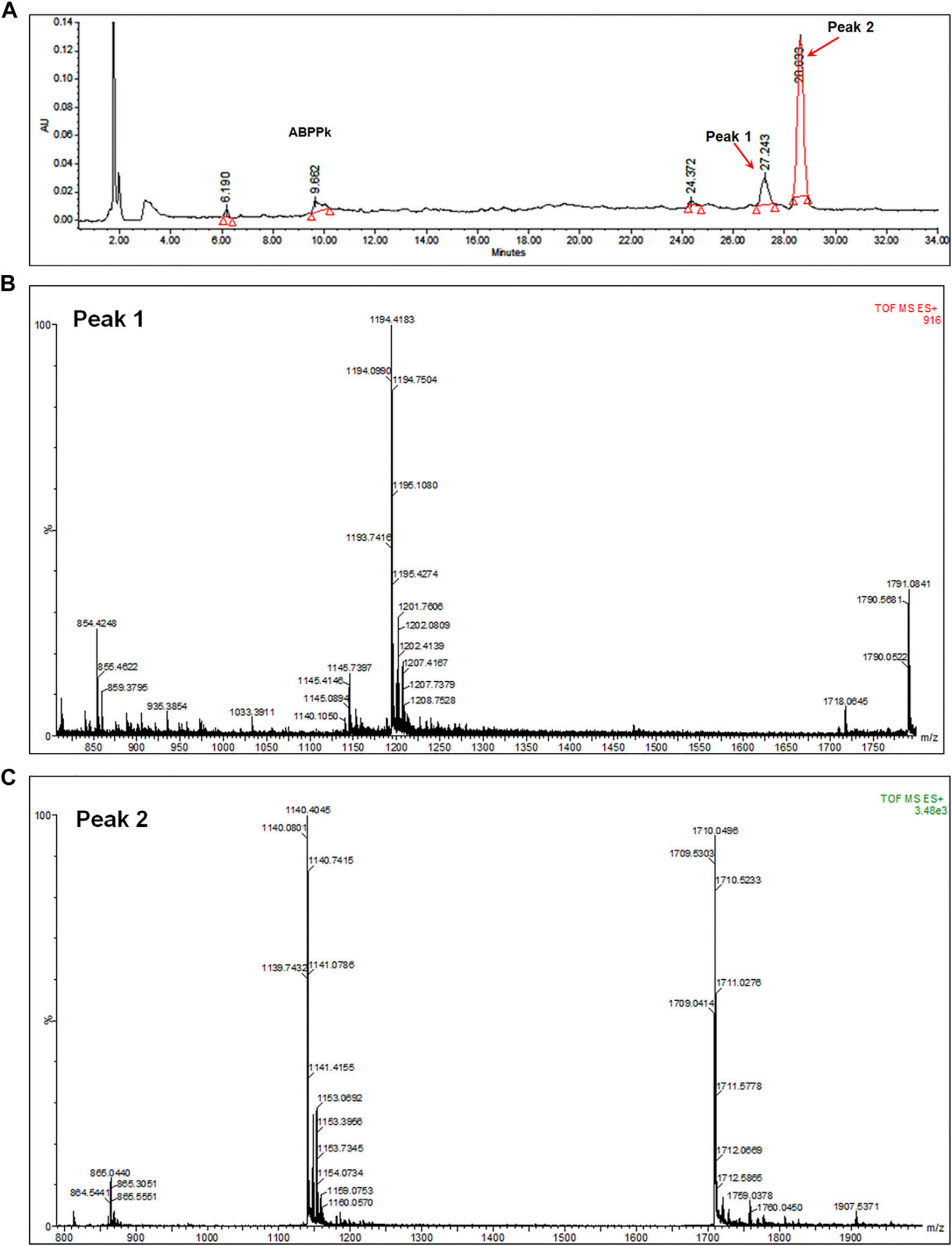
Chromatographic and mass spectrometry analysis of ABPPk. **(A)** Chromatographic analysis of ABPPk, monitored at 220 nm. Peaks numbered 1 and 2 are two peptides contained in ABPPk. **(B)** TOF-MS spectrometry of Peak 1, which was ionized to form trivalent ions with an average m/z of 1194.4 and divalent ions with an average m/z of 1791.1. The molecular weight of the polypeptide was about 3.58 kD. **(C)** TOF-MS spectrometry of Peak 2, which was ionized to form trivalent ions with an average m/z of 1140.4 and divalent ions with an average m/z of 1711.1. The molecular weight of the polypeptide was about 3.42 kD.

6 mg of *A. bidentate* polypeptide fraction k (ABPPk) was isolated from the 4 kg roots of *A. bidentate*. The extraction flowchart was shown in Supplementary Figure S1. The chromatographic and mass spectrometry analysis revealed that ABPPk contains two polypeptides with molecular weights of 3.58 kD and 3.42 kD, respectively, as shown in Figures 1B,C. Amino acid sequencing has been performed for the peptide with a molecular weight of 3.42 kD (Peak 2 in Figure 1A), which is the most abundant in ABPPk. The amino acid sequence and 3D structure of Peak 2 peptide was shown in Supplementary Figure S2.

### *A. bidentate* Polypeptide Fraction k Inhibited the Neurotoxicity of Aβ Oligomers by Alleviating the Neuroinflammatory Response

**FIGURE 2:**
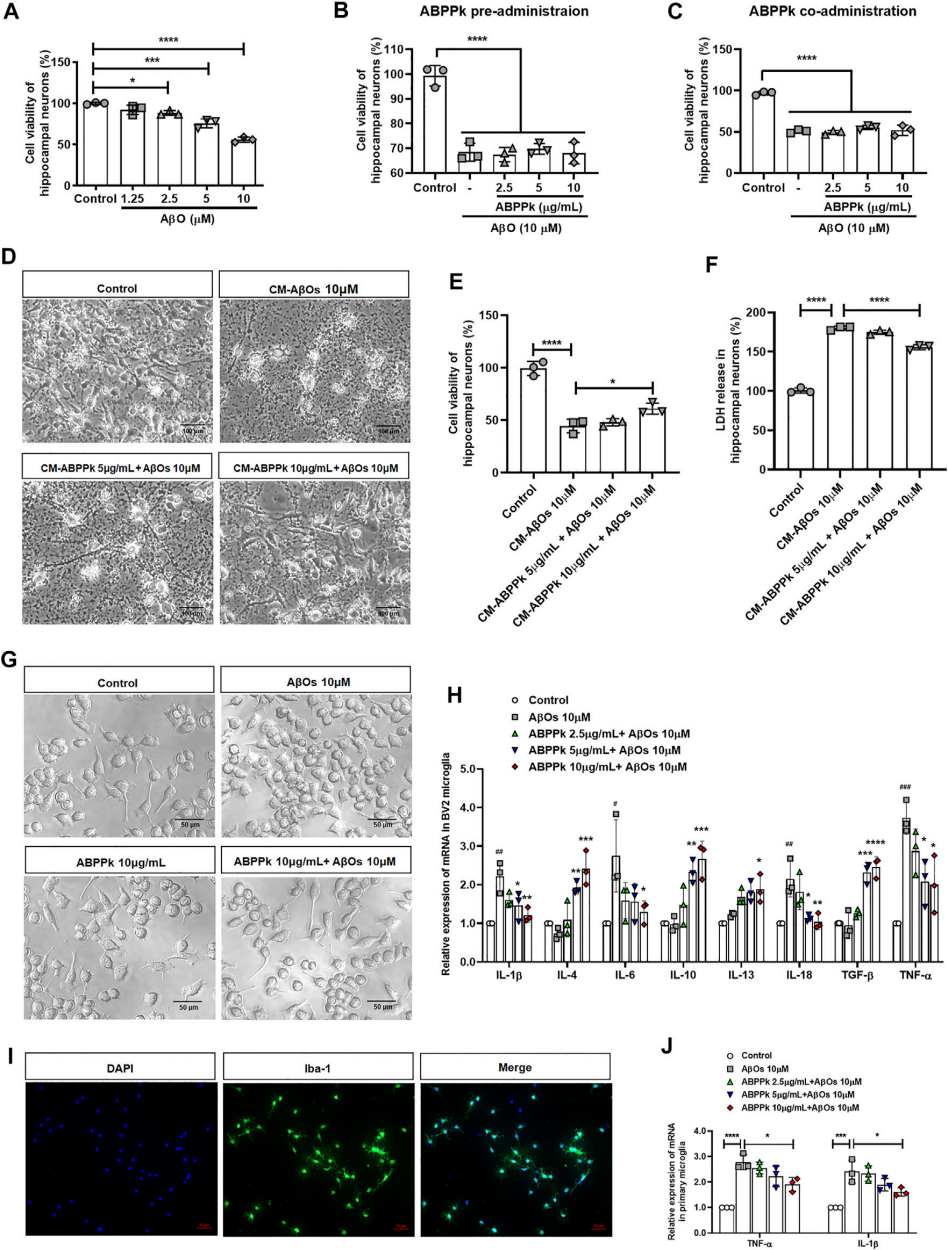
Effect of ABPPk on AβOs-induced neurotoxicity and neuroinflammatory response *in vitro*. **(A)** Effects of different concentrations of AβOs on the viability of hippocampal neurons. Data are shown as mean ± SD (*n* = 3). *p < 0.05, ***p < 0.001, ****p < 0.0001. **(B)** Effects of ABPPk pre-administration on AβOs-insulted hippocampal neuronal viability. Data are shown as mean ± SD (*n* = 3). ****p < 0.0001. **(C)** Effects of ABPPk co-administration on AβOs-insulted hippocampal neuronal viability. Data are shown as mean ± SD (*n* = 3). ****p < 0.0001. **(D)** Typical images of hippocampal neurons after treated with different BV2 microglia conditioned media (CM) for 24 h, acquired by phase-contrast microscope. Scale bar, 100 μm. **(E)** Cell viability of hippocampal neurons after incubation with BV2 microglia CMs for 24 h, determined by the CCK-8 assay. Data are expressed as mean ± SD (*n* = 3). *p < 0.05, ****p < 0.0001. **(F)** LDH release from hippocampal neurons after incubation with BV2 microglia CMs for 24 h, determined by the LDH assay. Data are expressed as mean ± SD (*n* = 3). ****p < 0.001. **(G)** The morphological changes of BV2 microglia after different treatments for 24 h, acquired by phase-contrast microscope. Scale bar, 50 μm. **(H)** The modulation of different concentrations of ABPPk on inflammatory cytokines in AβOs-stimulated BV2 microglia, measured by qPCR. Data are shown as mean ± SD (*n* = 3). #*p* < 0.05, ##*p* < 0.01, ###*p* < 0.001 vs. Control; *p < 0.05, **p < 0.01, ***p < 0.001, ****p < 0.0001 vs. AβOs 10 μM. **(I)** Representative images of cultured primary microglia examined by using anti-Iba-1 (green) antibody and DAPI (blue), acquired by Zeiss fluorescence microscopy. Scale bar, 50 μm. **(J)** The modulation of different concentrations of ABPPk on pro-inflammatory TNF-α and IL-1β in AβOs-stimulated primary microglia, measured by qPCR. Data are shown as mean ± SD (*n* = 3). *p < 0.05, ***p < 0.001, ****p < 0.0001.

We stimulated primary hippocampal neurons with different concentrations of AβOs. 10 μM AβOs decreased the viability of hippocampal neurons by about 40% (Figure 2A). Next, we investigated the protective effect of ABPPk on neurons by directly stimulating neurons with 10 μM AβOs. The results showed that neither pre-administration nor co-administration of ABPPk has robust effect on AβOs-induced neuronal damage (Figures 2B,C).

We wondered whether ABPPk could indirectly inhibit the neurotoxicity of AβOs by regulating microglial neuroinflammation. Therefore, the toxicity of BV2 microglia conditioned media (CM) on hippocampal neurons was examined. After cultured for 24 h, a massive neuronal bodies shrinkage and neurites collapse were observed in the CM-AβOs 10 μM cultured neurons with the viability decreased by 50%. Compared with CM-AβOs 10 μM, the viability of CM-ABPPk 10 μg/ml + AβOs 10 μM cultured neurons was significantly increased as shown in Figures 2D,E. The release of LDH was significantly increased in CM-AβOs 10 μM cultured neurons, while the release of LDH was significantly decreased in CM-ABPPk 10 μg/ml + AβOs 10 μM cultured neurons (Figure 2F).

The morphological changes of BV2 microglia induced by AβOs (10 μM) with or without ABPPk (10 μg/ml) were observed under phase contrast microscope. 10 μM AβOs-treated BV2 microglia revealed an amoeboid shape with enlarged cell body and shortened processes as shown in Figure 2G. 10 μg/ml ABPPk had no significant effect on the morphology of BV2 microglia. However, 10 μg/ml ABPPk pretreatment ameliorated the morphological changes of BV2 microglia caused by 10 μM AβOs, suggesting that ABPPk suppressed AβOs-induced microglia activation. We further investigated the effect of ABPPk on AβOs-induced inflammatory cytokines release in BV2 microglia. Relative mRNA expression levels of IL-1β, IL-4, IL-6, IL-10, IL-13, IL-18, TGF-β, and TNF-α were detected using qPCR. The quantitative data showed that the gene expression of pro-inflammatory IL-1β, IL-6, IL-18, and TNF-α were markedly enhanced by AβOs stimulation, whereas ABPPk pretreatment inhibited the effect of AβOs in a dose-dependent manner. It was noteworthy that anti-inflammatory IL-4, IL-10, IL-13, and TGF-β mRNA expressions were elevated by ABPPk dose-dependently, as shown in Figure 2H. The dose response studies of ABPPk on AβOs stimulated BV2 microglia were performed by the IL-1β ELISA kit. The IC50 of ABPPk was about 8 μg/ml fit by GraphPad 8.0 software. The results were presented in Supplementary Figure S3. We also used primary cultured microglia to confirm the inhibitory effect of ABPPk on AβOs-induced inflammation. The identification of primary microglia by Iba-1 immunostaining was shown in Figure 2J. AβOs (10 μM) also induced the TNF-α and IL-1β mRNAs up-regulation in the primary microglia, while pretreatment with ABPPk suppressed the expressions of TNF-α and IL-1β mRNAs, especially at the concentration of 10 μg/ml (Figure 2J). These results indicated that ABPPk reduces AβOs-induced neurotoxicity most probably through regulating microglial inflammation.

### *A. bidentate* Polypeptide Fraction k Inhibited the Activation of NF-κB and NLRP3 in Aβ Oligomers-Stimulated BV2 Microglia

**FIGURE 3:**
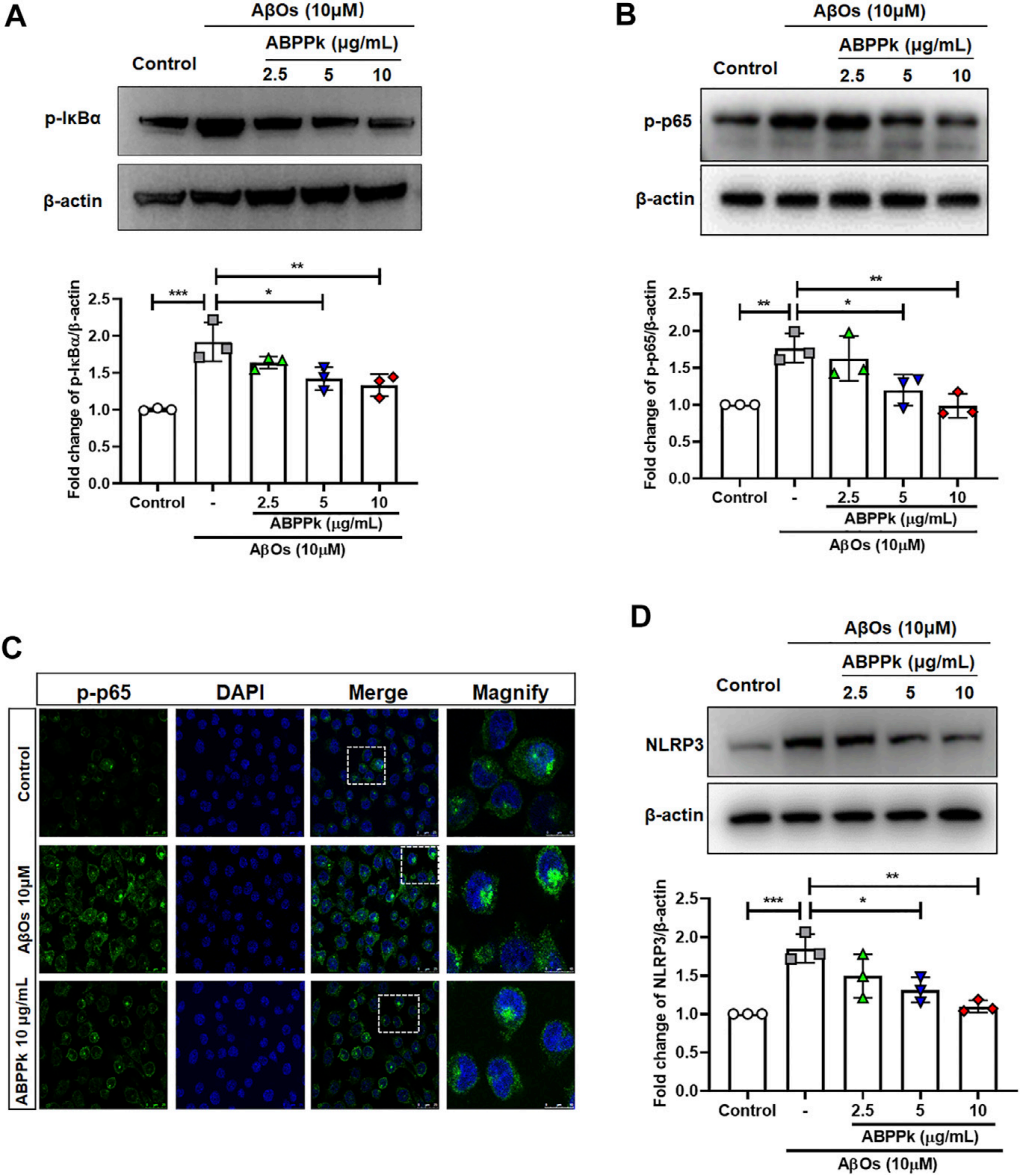
Effect of ABPPk on AβOs-induced NF-κB and NLRP3 activation in BV2 microglia. **(A)** Representative Western blot image and quantitative analysis of phosphorylated IκBα (p-IκBα). β-actin was used as an internal control. Bar graph showing the fold changes of p-IκBα relative to β-actin. Data are expressed as mean ± SD (*n* = 3). *p < 0.05, **p < 0.01, ***p < 0.001. **(B)** Representative Western blot image and quantitative analysis of phosphorylated NF-κB (p-p65). β-actin was used as an internal control. Bar graph showing the fold changes of p-p65 relative to β-actin. Data are expressed as mean ± SD (*n* = 3). *p < 0.05, **p < 0.01. **(C)** Representative images of NF-κB nuclear translocation examined by using anti-phosphor-NF-κB (p-p65) (green) antibody and DAPI (blue), acquired by Leica confocal microscopy. Scale bar for the merge column, 25 μm. The magnify column is a magnification of the white dotted box in the merge column with a scale bar of 10 μm. **(D)** Representative Western blot images and quantitative analysis for NLRP3. β-actin was used as an internal control. Bar graph showing the fold changes of NLRP3 relative to β-actin. Data are expressed as mean ± SD (*n* = 3). *p < 0.05, **p < 0.01, ***p < 0.001.

Aβ stimulates microglia to activate NF-κB and produce NLRP3 inflammasomes (Bauernfeind et al., 2009). NF-κB is a recognized major regulator of inflammatory cytokine genes, which is present in the cytoplasm in an inactive state complexed with the inhibitory IκB proteins. IκBα inhibits nuclear translocation of NF-κB by retaining it in the cytosol. Activation of IκBα via phosphorylation at Ser32 and Ser36 followed by proteasome-mediated degradation, results in the release and nuclear translocation of active NF-κB. We examined the effects of ABPPk on AβOs-induced phosphorylation of IκBα and activation of NF-κB by western blot. 10 μM of AβOs significantly increased the phosphorylation levels of IκBα and NF-κB. However, pretreatment with 5 and 10 μg/ml ABPPk effectively inhibited the phosphorylation of IκB-α and NF-κB p65 (Figures 3A,B). Confocal microscopy was used to further determine whether ABPPk pretreatment affects the nuclear translocation of NF-κB. The p65 subunit of NF-κB was present predominantly in the cytoplasm under normal condition. AβOs increased the nuclear translocation of the p-p65 subunit, however, pretreatment with 10 μg/ml ABPPk attenuated the effect of AβOs (Figure 3C), indicating the inhibiting role of ABPPk on the transcriptional activity of NF-κB. The expression level of NLRP3 was significantly up-regulated in AβOs-stimulated BV2 microglia, but ABPPk pretreatment down-regulated the level of NLRP3 dose-dependently (Figure 3D).

### *A. bidentate* Polypeptide Fraction k Improved Locomotion Activity and Ameliorated Memory Deficits in Aβ Oligomers-Injected Mice

**FIGURE 4:**
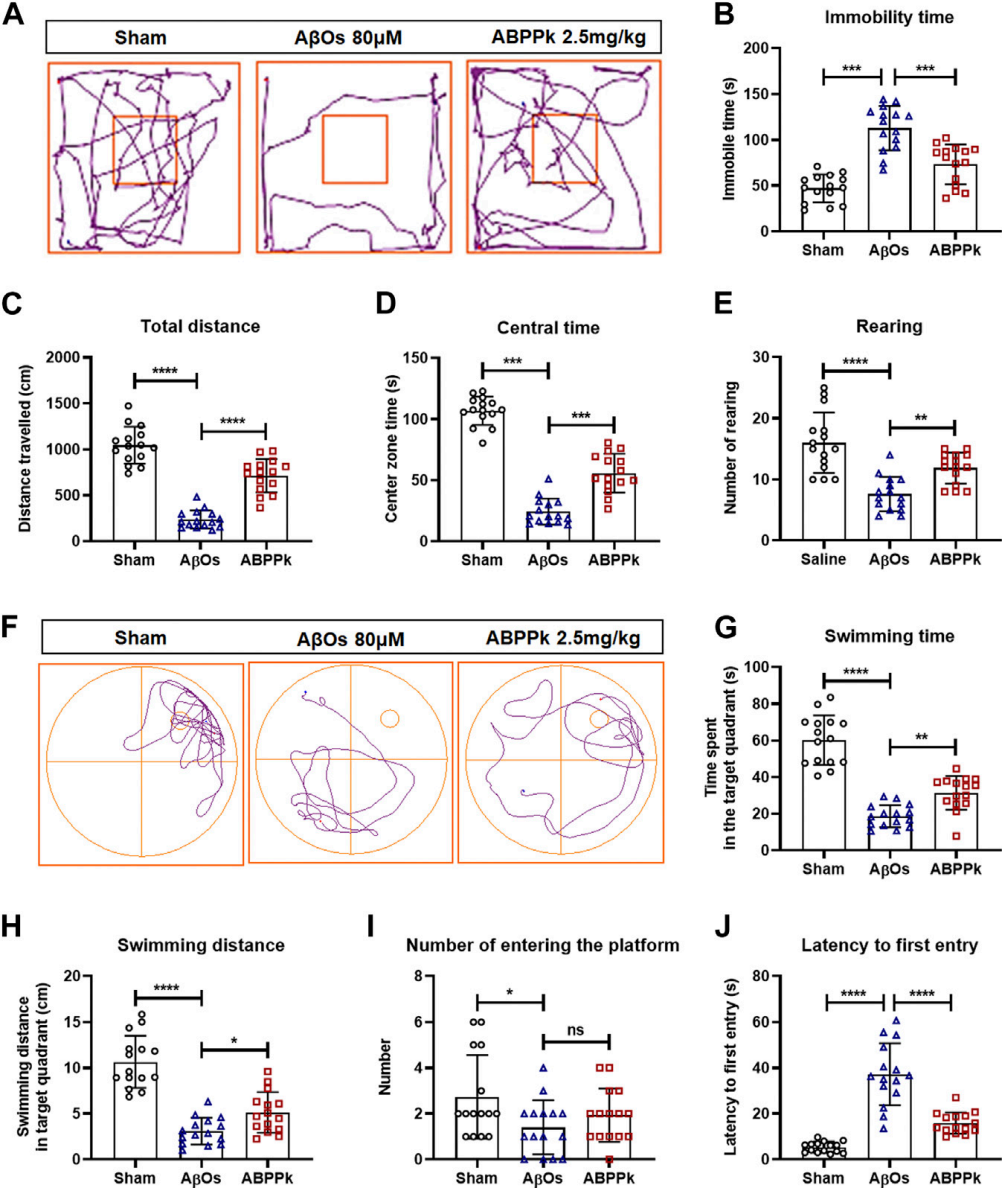
Effect of ABPPk on locomotion activity and memory deficits in AβOs-injected mice. **(A–E)** Open-field test. The representative path tracings of each group **(A)**, the immobility time **(B)**, the total distance **(C)**, the center zone time **(D)**, and the number of rearing **(E)**. **(F–J)** Morris water maze test. The representative path tracings of each group **(F)**, the time **(G)** and distance **(H)** swimming in the target quadrant, the number of crossing through the platform **(I)**, and the latency to first entry into the target quadrant **(J)**. Data are expressed as mean ± SD (*n* = 15). *p < 0.05, **p < 0.01, ***p < 0.001, ****p < 0.0001. ns means no significance.

Locomotor activity of mice was assessed using open field test at 24 h after AβOs injection to mice. The typical track in the open field of each group of mice was shown in Figure 4A. AβOs (80 μM) significantly prolonged the immobility time (Figure 4B), reduced the total travelling distance in the open field (Figure 4C) and the time spent in the central area (Figure 4D), and decreased the frequency with which the mice stood on their hind legs in the field (number of rearing) (Figure 4E), indicating that AβOs injection induced lower locomotor activity in mice. However, pre-administration of 2.5 mg/kg of ABPPk significantly increased the total travelling distance and the duration of time spent in the central square, reduced the time of immobility and increased the rearing number significantly compared with AβOs-injected mice (Figures 4A–E).

After open field test, mice were subjected to Morris water maze test to evaluate the effect of ABPPk on their memory ability. The three groups of mice were subjected to a probe trial with the platform removed, the typical swimming track of each group of mice was shown in Figure 4F. Mice in the AβOs group had a remarkably decreased swimming time and distance in the target quadrant as well as a reduced frequency of entries to the original platform position (Figures 4G–I). In contrast, mice in the ABPPk group spent more time with longer distance in target quadrant in comparison with the AβOs group (Figures 4G,H). The latency to enter the target quadrant was higher in the AβOs group than in the sham group, while the latency was significantly lower in the ABPPk group than in the AβOs group (Figure 4J). Behavioral test showed that ABPPk improves the locomotor activity and memory function insulted by AβOs injection.

### *A. bidentate* Polypeptide Fraction k Reduced the Degeneration of Hippocampal Neurons in Aβ Oligomers-Injected Mice Brain

**FIGURE 5:**
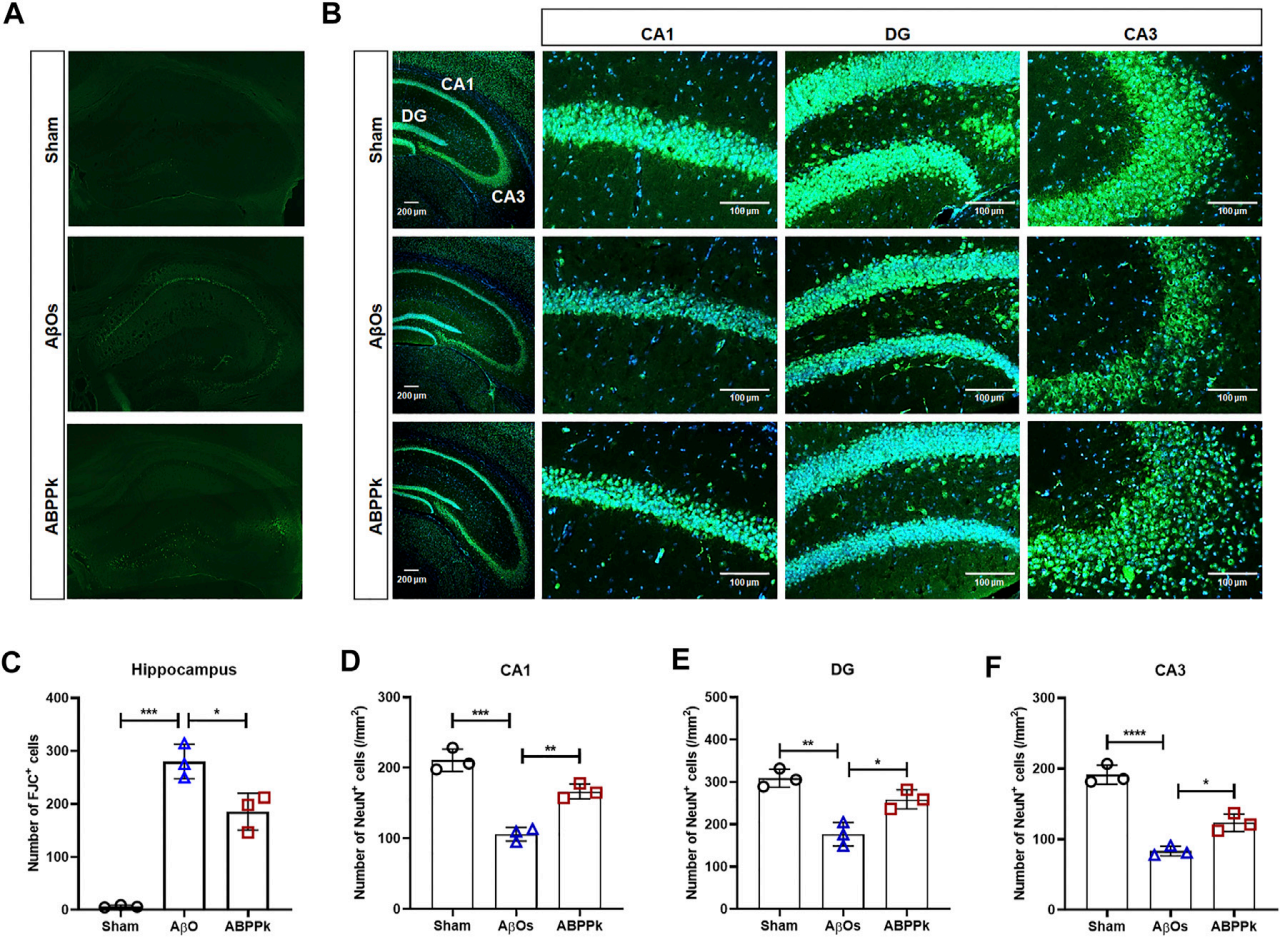
Effect of ABPPk on AβOs-induced hippocampal neuronal loss in mice. **(A)** Representative images of the hippocampus examined by Fluor-Jade C (FJC) staining, acquired by Zeiss fluorescence microscopy. **(B)** Representative images of NeuN-positive neurons in the hippocampus examined by using anti-NeuN (green) antibody and DAPI (blue), acquired by Zeiss fluorescence microscopy. Scale bar for the overall picture of the hippocampus, 200 μm. Scale bar for the picture of CA1, DG, and CA3 regions, 100 μm. **(C)** Quantitative analysis of FJC-positive cells in the hippocampus of each group. Data are expressed as mean ± SD (*n* = 3). *p < 0.05, ***p < 0.001. **(D–F)** Quantitative analysis of NeuN-positive cells in the CA1 region **(D)**, DG region **(E)**, and CA3 region **(F)** of each group. Data are expressed as mean ± SD (*n* = 3). *p < 0.05, **p < 0.01, ***p < 0.001, ****p < 0.0001.

Depression-like behavior and cognitive deficits in AD are closely related to neuronal damage caused by AβOs. After behavior test, Fluor-Jade C (FJC) staining of brain slices was performed to evaluate the neuroprotective effect of ABPPk against AβOs-induced hippocampal neurodegeneration. AβOs (80 μM) significantly led to the neurodegeneration of hippocampal neurons, but 2.5 mg/kg ABPPk pre-administration alleviated the neurodegeneration as shown in Figures 5A,C.

We also measured the change in the density of NeuN-positive neurons in the hippocampus of AβOs-injected brain by immunohistochemical staining for NeuN. The result confirmed that 2.5 mg/kg ABPPk pre-administration prevented the loss of NeuN-positive neurons in the hippocampus (Figure 5B). The density analysis showed that the number of NeuN-positive cells of hippocampal cornu ammonis 1 (CA1), dentate gyrus (DG), and cornu ammonis 3 (CA3) regions in AβOs group was significantly lower than that in the sham group. Compared with AβOs group, ABPPk significantly increased the density of NeuN-positive cells (Figures 5D–F). These results indicated that ABPPk pre-administration prevented hippocampal neuronal degeneration in AβOs-injected mice.

### *A. bidentate* Polypeptide Fraction k Inhibited the Activation of Microglia and Regulated the Inflammatory Cytokines Release in Aβ Oligomers-Injected Mice Brain

**FIGURE 6:**
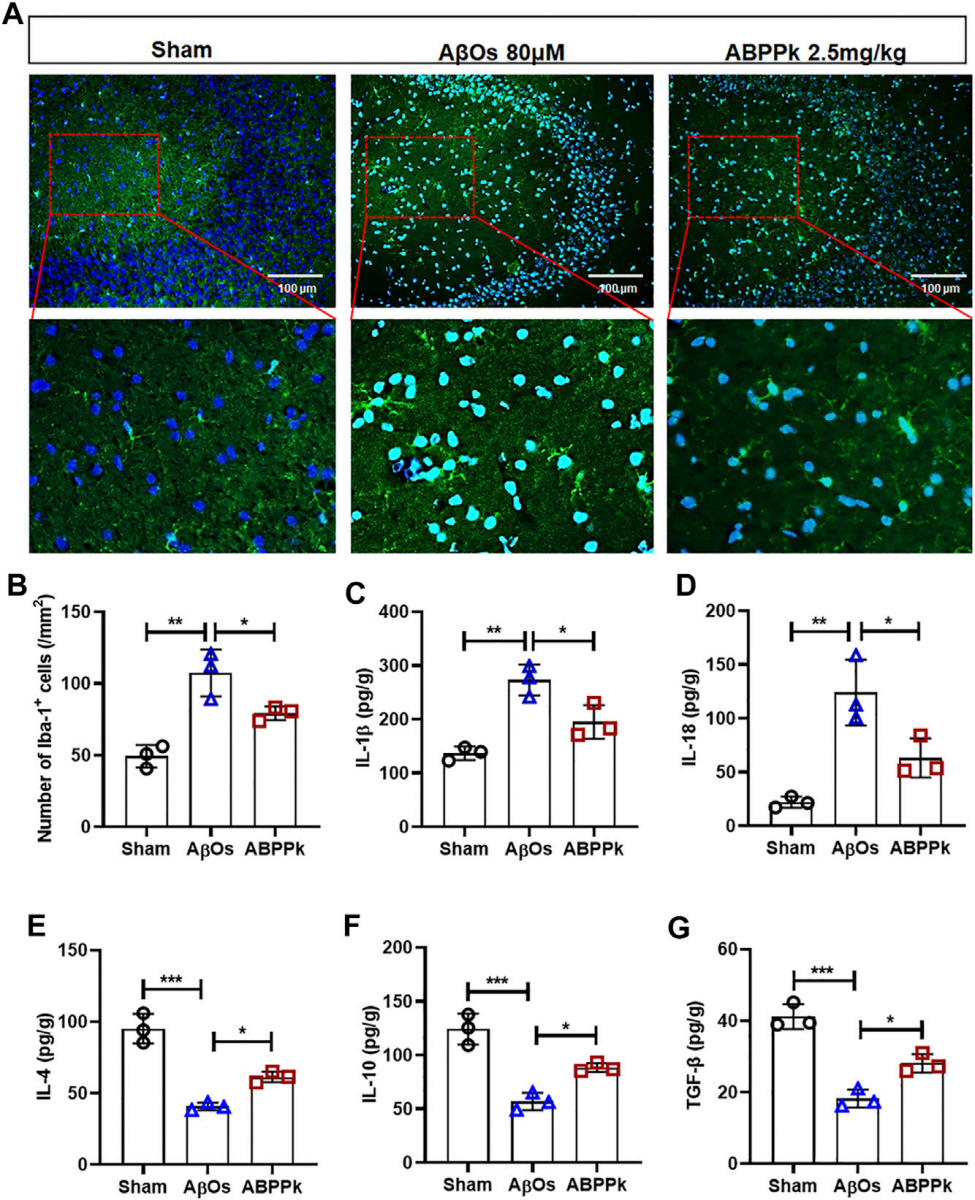
Effect of ABPPk on AβOs-induced neuroinflammation in mice. **(A)** Representative images of Iba-1-positive microglia in the hippocampus CA3 region examined by using anti-Iba-1 (green) antibody and DAPI (blue), acquired by Zeiss fluorescence microscopy. Scale bar for the upper line of images, 100 μm. The bottom line of images is a partial enlargement of the red dotted box in the upper line. **(B)** Quantitative analysis of Iba-1-positive cells in the CA3 region of each group. Data are expressed as mean ± SD (*n* = 3). *p < 0.05, **p < 0.01. **(C–G)** The content of IL-1β **(C)**, IL-18 **(D)**, IL-4 **(E)**, IL-10 **(F)**, and TGF-β **(G)** in the supernatant of brain tissue, determined by ELISA kits. Data are expressed as mean ± SD (*n* = 3). *p < 0.05, **p < 0.01, ***p < 0.001.

Aβ can not only directly cause neuronal damage, but also activate microglia to release inflammatory cytokines and affect the surrounding brain tissue. We observed the activation of microglia in hippocampal CA3 region. The number of Iba-1 positive cells per field in the hippocampal CA3 region was significantly reduced in ABPPk group compared with that in the AβOs group (Figures 6A,B). ELISA results showed that the expression levels of pro-inflammatory IL-1β and IL-18 were significantly decreased in ABPPk group compared with AβOs group (Figures 6C,D), while the levels of anti-inflammatory IL-4, IL-10, and TGF-β in the ABPPk group were significantly higher than those in the AβOs injection group (Figures 6E–G), suggesting that ABPPk pre-administration regulates the release of inflammatory cytokines in AβOs-injected mice.

### *A. bidentate* Polypeptide Fraction k Inhibited the Activation of NF-κB and NLRP3 in Aβ Oligomers-Injected Mice Brain

**FIGURE 7:**
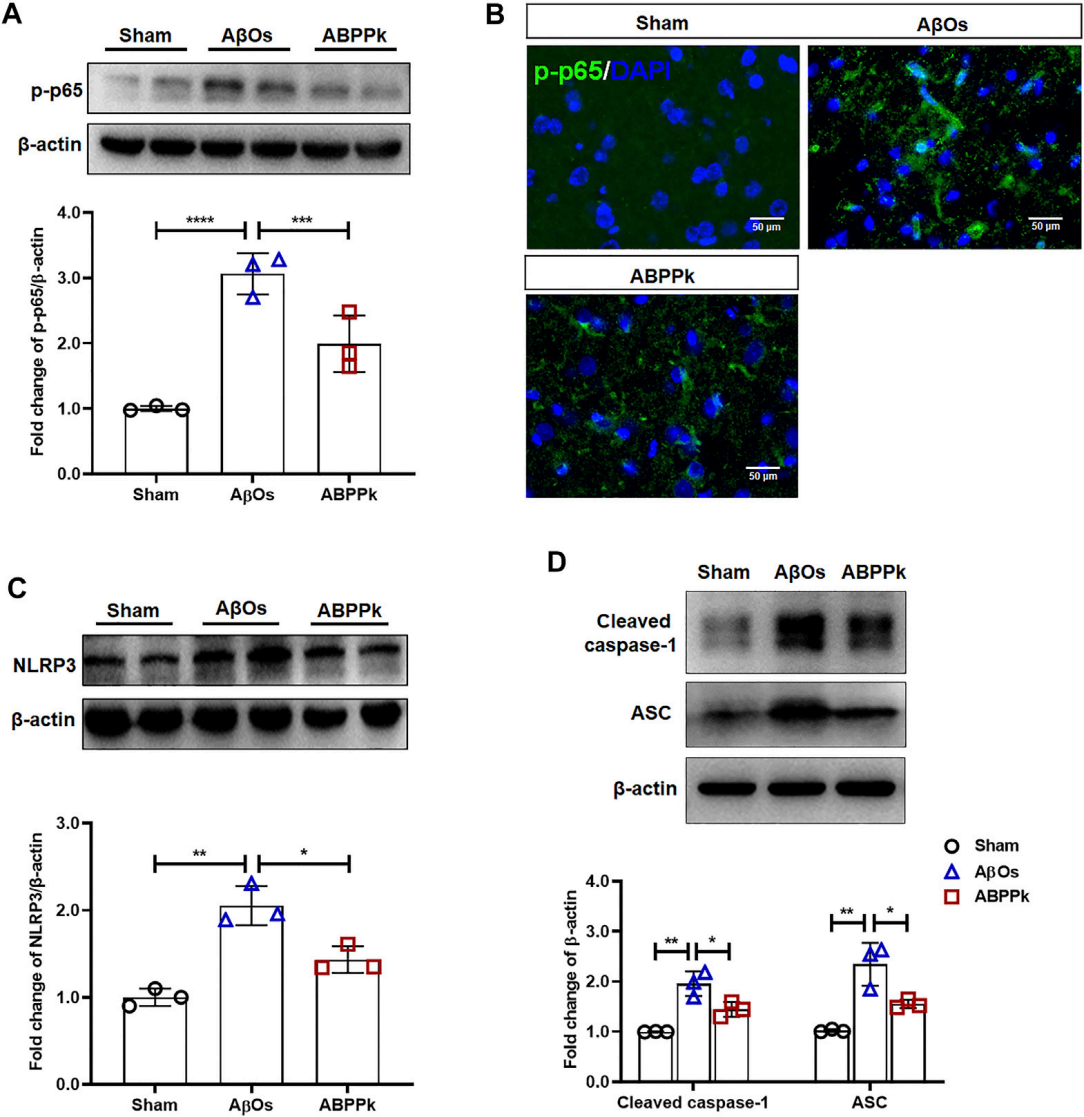
Effect of ABPPk on NF-κB and NLRP3 activation in AβOs-injected mice brain. **(A)** Representative Western blot image and quantitative analysis of phosphorylated NF-κB (p-p65). β-actin was used as an internal control. Bar graph showing the fold changes of p-p65 relative to β-actin. Data are expressed as mean ± SD (*n* = 3). ***p < 0.001, ****p < 0.0001. **(B)** Representative images of NF-κB p65 nuclear translocation in the brain examined by using anti-phosphor-NF-κB (p65) (green) antibody and DAPI (blue), acquired by immunofluorescence microscopy. Scale bar, 50 μm. **(C)** Representative Western blot image and quantitative analysis of NLRP3 in the brain. β-actin was used as an internal control. Bar graph showing the fold changes of NLRP3 relative to β-actin. Data are expressed as mean ± SD (*n* = 3). *p < 0.05, **p < 0.01. **(D)** Representative Western blot image and quantitative analysis of cleaved caspase-1 and ASC in the brain. β-actin was used as an internal control. Bar graph showing the fold changes of Cleaved caspase-1 and ASC relative to β-actin. Data are expressed as mean ± SD (*n* = 3). *p < 0.05, **p < 0.01.

Western blot analysis showed that intracerebroventricular injection of AβOs resulted in a significant increase in the phosphorylation level of NF-κB p65, while ABPPk inhibited this up-regulation (Figure 7A). Immunofluorescence histochemical staining of phosphorylated NF-κB p65 showed that nuclear translocation of NF-κB was less obvious in ABPPk group than in AβOs group (Figure 7B), which was consistent with the results of *in vitro* experiments. It is known that inflammasomes are generally assembled as multiple protein complexes in the cytoplasm, which mainly contain NOD-like receptors (NLRs), apoptosis-associated speck-like protein containing a CARD (ASC), and pro-caspase 1. In our study, 80 μM AβOs i.c.v. injection significantly increased the expression levels of NLRP3, ASC and cleaved-caspase1 in the brain of mice, while 2.5 mg/kg ABPPk pre-administration robustly inhibited their expressions (Figures 7C,D).

### *A. bidentate* Polypeptide Fraction k Suppressed M1-type Polarization and Promoted M2-type Polarization in Activated BV2 Microglia

**FIGURE 8:**
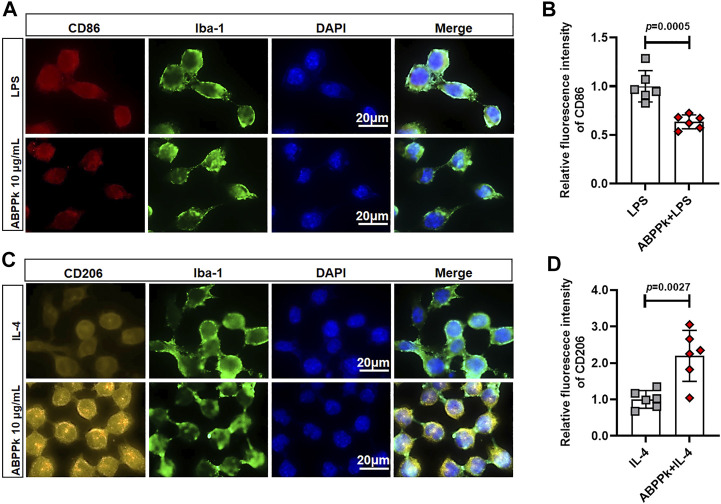
Effect of ABPPk on the polarization of BV2 microglia. **(A)** Representative images of LPS-induced M1-phenotype microglia in each group examined by using anti-CD86 (red), anti-Iba-1 (green) antibodies and DAPI (blue), acquired by Zeiss fluorescence microscopy. Scale bar, 20 μm. **(B)** Relative fluorescence intensity analysis for LPS-induced M1-phenotype in the presence or absence of ABPPk pretreatment. Data are expressed as mean ± SD (*n* = 6). **(C)** Representative images of IL-4-induced M2-phenotype microglia in each group examined by using anti-CD206 (orange), anti-Iba-1 (green) antibodies and DAPI (blue), acquired by Zeiss fluorescence microscopy. Scale bar, 20 μm. **(D)** Relative fluorescence intensity analysis for IL-4-induced M2-phenotype in the presence or absence of ABPPk pretreatment. Data are expressed as mean ± SD (*n* = 6).

Different subtypes of microglia express classical activated M1 phenotype markers such as CD86 and alternative activated M2 phenotype markers such as CD206 (Franco and Fernández-Suárez, 2015). Given that ABPPk can inhibit the expression of pro-inflammatory cytokines and promote the expression of anti-inflammatory cytokines, we carried out a polarization experiment to determine whether ABPPk could regulate the polarization of BV2 microglia. BV2 microglia were cultivated in medium containing microglial polarization stimuli (LPS or IL-4) with or without 10 μg/ml ABPPk pretreatment. After 24 h, immunocytochemistry results showed that pro-inflammatory LPS (1 μg/ml) induced CD86 positive phenotype in BV2 microglia, while ABPPk pretreatment for 30 min significantly inhibited M1 polarization (Figures 8A,B). Anti-inflammatory IL-4 (0.1 μg/ml) induced CD206 positive M2 phenotype in BV2 microglia, while ABPPk pretreatment significantly increased M2 polarization (Figures 8C,D). The results indicated that ABPPk inhibited M1 polarization and promoted M2 polarization in BV2 microglia.

### *A. bidentate* Polypeptide Fraction k Regulated the Polarization of Microglia Towards M2-Phenotype by Activating Autophagy in BV2 Microglia

**FIGURE 9:**
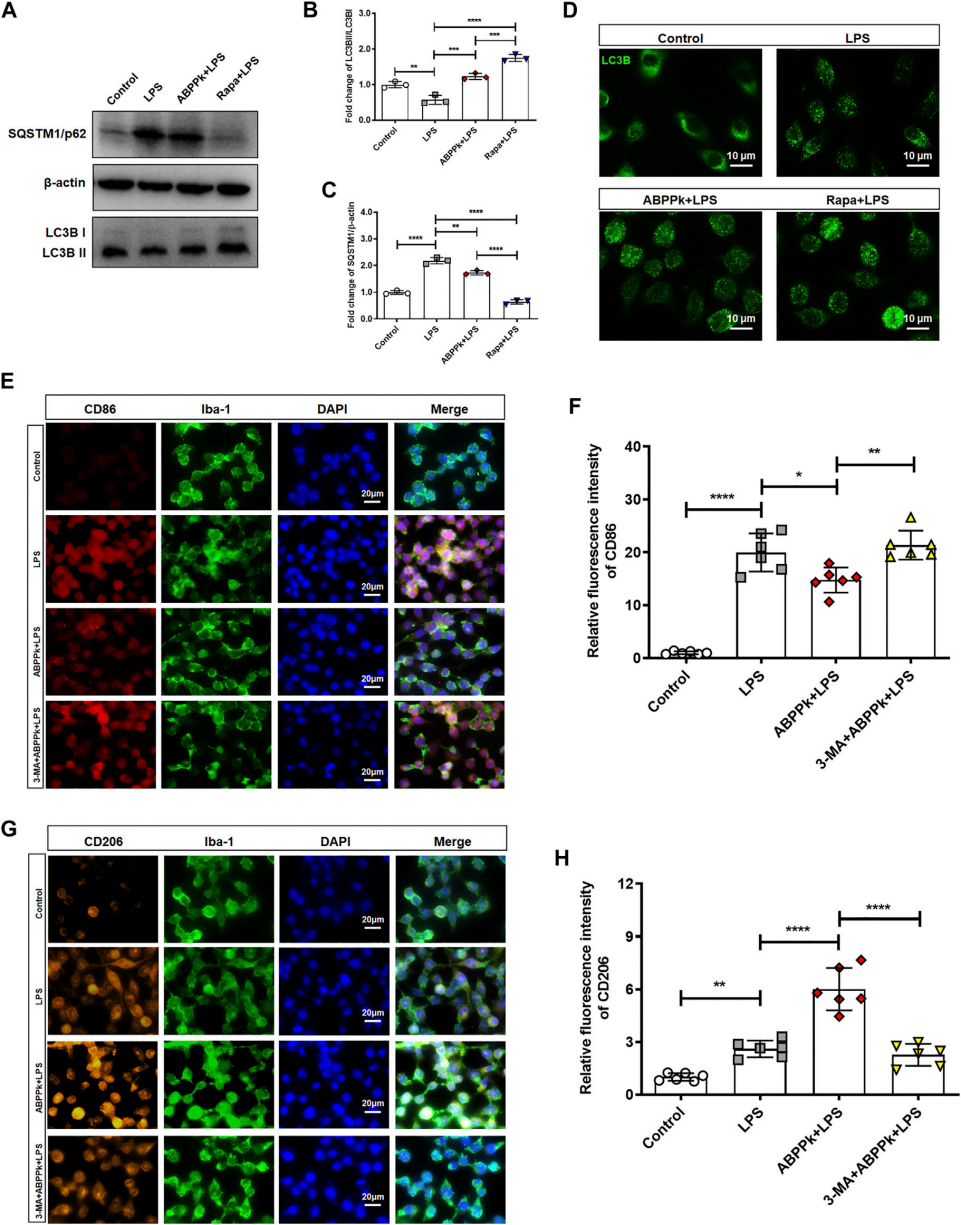
Effect of ABPPk on autophagy and polarization of LPS-activated BV2 microglia. **(A)** Representative Western blot images for SQSTM1/p62 and LC3B. β-actin was used as an internal control. **(B)** Quantification of blots showing the fold changes of LC3B-II to LC3B-I. Data are expressed as mean ± SD (*n* = 3). **p < 0.01, ***p < 0.001, ****p < 0.0001. **(C)** Quantification of blots showing the fold changes of SQSTM1/p62 to β-actin. Data are expressed as mean ± SD (*n* = 3). **p < 0.01, ****p < 0.0001. **(D)** Representative images of autophagic flux verified by using anti-LC3B-II/I (green) antibody, acquired by Zeiss fluorescence microscopy. Scale bar, 10 μm. **(E)** Representative images of M1-phenotype microglia in each group examined by using anti-CD86 (red), anti-Iba-1 (green) antibodies and DAPI (blue), acquired by Zeiss fluorescence microscopy. Scale bar, 20 μm. **(F)** Relative fluorescence intensity analysis for LPS-induced M1-phenotype. Data are expressed as mean ± SD (*n* = 6). *p < 0.05, **p < 0.01, ****p < 0.0001. **(G)** Representative images of M2-phenotype microglia in each group examined by using anti-CD206 (orange), anti-Iba-1 (green) antibodies and DAPI (blue), acquired by Zeiss fluorescence microscopy. Scale bar, 20 μm. **(H)** Relative fluorescence intensity analysis for LPS-induced M2-phenotype. Data are expressed as mean ± SD (*n* = 6). **p < 0.01, ****p < 0.0001.

Autophagy can control the polarization state of microglia, regulate the inflammatory response, and affect the survival of neurons in neurodegeneration diseases (Liu et al., 2015; Jin et al., 2018). After pretreatment with 10 μg/ml ABPPk or 100 nM rapamycin (autophagy activator, abbreviated as Rapa) for 30 min, BV2 microglia were treated with 1 μg/ml LPS for 24 h to observe the effect of ABPPk on the autophagy of BV2 microglia. Western blot results showed that the expression of autophagosome marker, SQSTM1/p62, was up-regulated, and the autophagy flux (ratio of LC3B-II/I) was reduced by LPS, indicating that 1 μg/ml LPS stimulation for 24 h inhibited the autophagy in BV2 microglia. However, 10 μg/ml ABPPk pretreatment down-regulated the expression of SQSTM1/p62 and increased the autophagy flux of LC3B-II/I, which was similar to that of Rapa (Figures 9A–C). In the process of autophagy formation, LC3B-I located in the cytoplasm will be modified and processed by the ubiquitin-like system such as Atg 7 and Atg 3 to produce LC3B-II and translocation to the membrane of the autophagosome (Sarkar et al., 2007). By immunofluorescence chemical staining, we observed enhanced LC3B translocation fluorescence signal in both the ABPPk pre-treated and the Rapa pre-treated cells (Figure 9D), which was consistent with the autophagy flux detected by Western blot. These results indicated that ABPPk enhanced the LPS-inhibited microglia autophagy.

In order to further verify the effect of autophagy on polarization, we used the autophagy inhibitor 3-Methyladenine (Abbreviated as 3-MA) to inhibit the autophagy before ABPPk and/or LPS treatment of BV2 microglia. Immunofluorescence cytochemistry results revealed that, compared with the LPS group, the fluorescence intensity of CD86-positive M1-type cells was significantly decreased in ABPPk group (Figures 9E,F), while the fluorescence intensity of CD206-positive M2-type cells was significantly increased (Figures 9G,H). However, 5 μM 3-MA pretreatment for 3 h eliminated the regulatory effect of ABPPk on microglia polarization, suggesting that ABPPk may probably regulate microglia polarization through the activation of autophagy.

### *A. bidentate* Polypeptide Fraction k Enhanced the Aβ Oligomers-Impaired Autophagy and Promoted M2-type Polarization of BV2 Microglia

**FIGURE 10:**
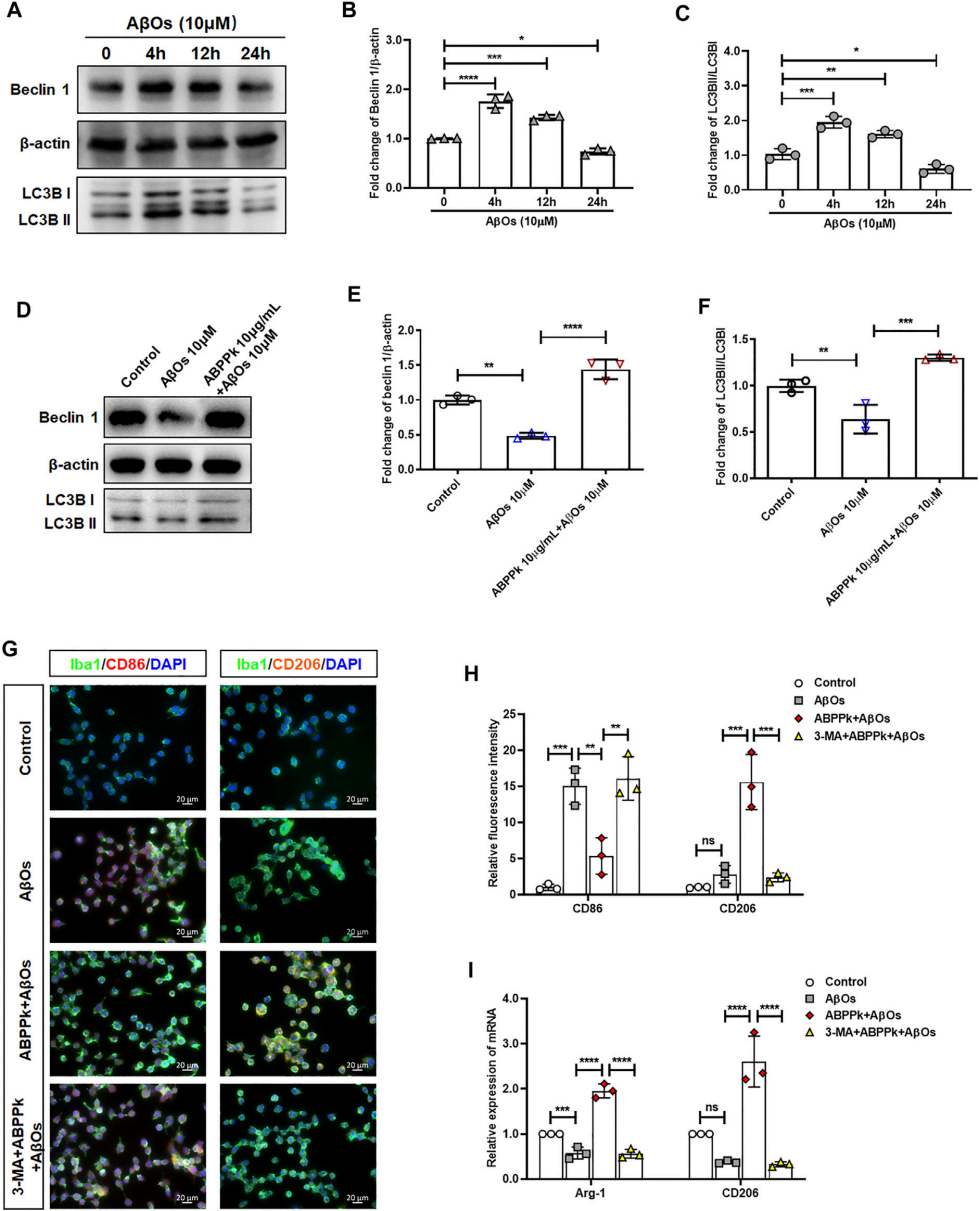
Effect of ABPPk on autophagy and polarization in AβOs-stimulated BV2 microglia. **(A)** Representative Western blot images for Beclin 1 and LC3B. β-actin was used as an internal control. **(B)** Quantification of blots showing the fold changes of Beclin to β-actin. Data are expressed as mean ± SD (*n* = 3). *p < 0.05, ***p < 0.001, ****p < 0.0001. **(C)** Quantification of blots showing the fold changes of LC3B-II to LC3B-I. Data are expressed as mean ± SD (*n* = 3). *p < 0.05, **p < 0.01, ***p < 0.001. **(D)** Representative Western blot images for Beclin 1 and LC3B. β-actin was used as an internal control. **(E)** Quantification of blots showing the fold changes of Beclin 1 to β-actin. Data are expressed as mean ± SD (*n* = 3). **p < 0.01, ****p < 0.0001. **(F)** Quantification of blots showing the fold changes of LC3B-II to LC3B-I. Data are expressed as mean ± SD (*n* = 3). **p < 0.01, ***p < 0.001. **(G)** Representative images of M1-or M2-phenotype microglia in each group examined by using anti-CD86 (red), anti-CD206 (orange), anti-Iba-1 (green) antibodies and DAPI (blue), acquired by Zeiss fluorescence microscopy. Scale bar, 20 μm. **(H)** Relative fluorescence intensity analysis for CD86 positive (M1-type) and CD206 positive (M2-type) cells in AβOs-stimulated BV2 microglia. Data are expressed as mean ± SD (*n* = 3). **p < 0.01, ***p < 0.001, ns means no significance. **(I)** The expression levels of M2-phenotype markers Arg-1 and CD206 in each group, measured by q-PCR. Data are shown as mean ± SD (*n* = 3). ***p < 0.001, ****p < 0.0001, ns means no significance.

Aβ may be engulfed by activated microglia or degraded by activated microglia autophagy highly in a Beclin 1-dependent way (Lucin et al., 2013). When autophagy machinery is impaired by chronic stimulation, the degradation of Aβ is hindered (Solé-Domènech et al., 2016). We investigated the effects of 10 μM AβOs on microglia autophagy. The results showed that AβOs stimulation for 4 and 12 h enhanced expression of Beclin 1 and the autophagy flux (LC3BII/I ratio) of microglia, while AβOs stimulation for 24 h resulted in a decrease of Beclin 1 expression and autophagy flux, suggesting an impaired microglia autophagy (Figures 10A–C). After pretreatment with 10 μg/ml ABPPk for 30 min, microglia were treated with 10 μM AβOs for 24 h. The Western blot results showed that ABPPk pretreatment significantly enhanced the AβOs-impaired autophagy (Figures 10D–F). Immunofluorescent staining results of CD86 positive or CD206 positive BV2 microglia showed that 10 μg/ml ABPPk pretreatment significantly inhibited the 10 μM AβOs-induced M1-type polarization and promoted the M2-type polarization, but after pretreated with 5 μM 3-MA for 3 h, the regulatory effect of ABPPk on microglia polarization disappeared (Figures 10G,H). Meanwhile, the mRNA levels of two typical M2-phenotype markers (Arg1 and CD206) of AβOs-stimulated BV2 microglia were detected by qPCR. The results showed that 10 μg/ml ABPPk significantly increased the mRNA levels of Arg1 and CD206 in AβOs-stimulated BV2 microglia, but this promotion could be abolished by pretreatment with 3-MA (Figure 10I), suggesting that ABPPk may promote M2-type polarization of microglia by promoting or maintaining autophagy.

## Discussion

**FIGURE 11:**
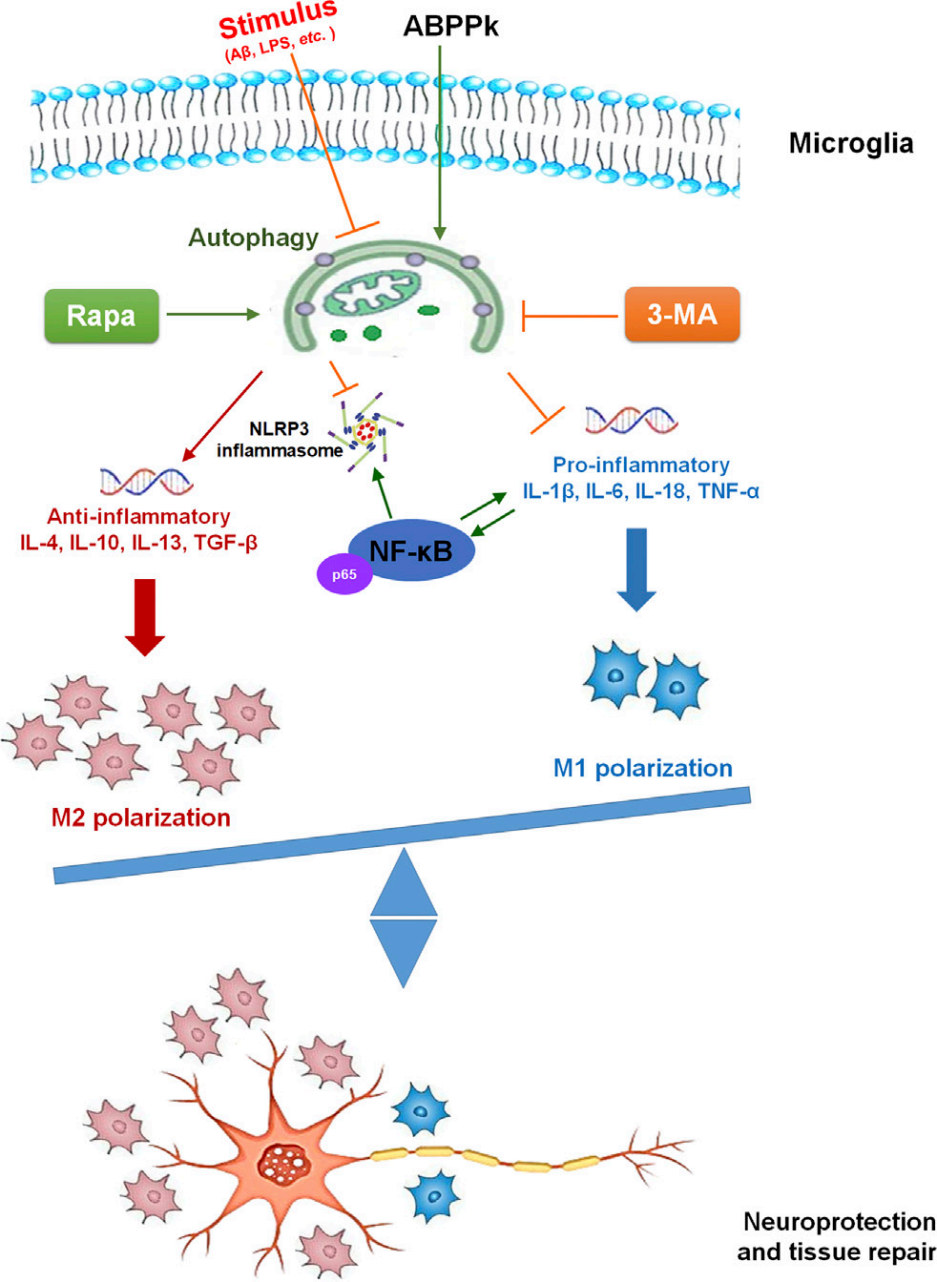
The mechanism diagram of ABPPk exerting neuroprotective effect.

The present study demonstrated the anti-inflammatory effect of ABPPk on AβOs-induced neuroinflammation by a set of experiments. *In vitro*, pretreatment of 5–10 μg/ml ABPPk inhibited the neuronal toxicity of AβOs by alleviating the neuroinflammatory response, reducing NF-κB transcriptional activity, and suppressing NLRP3 expression in BV2 microglia. *In vivo*, pre-administration of ABPPk at the dosage of 2.5 mg/kg improved locomotion activity, ameliorated memory deficits, decreased the hippocampal neuronal loss in AβOs intracerebroventricular injection mice. Polarization experiment suggested that ABPPk may regulate neuroinflammation via facilitating a M1 to M2 shift of microglia probably through enhancing autophagy. These results suggested that ABPPk has a potential protective effect against AβOs-induced neuroinflammation mainly through the regulation of microglia polarization (Figure 11).

Aβ accumulation, tau pathology, and neuroinflammation are typical hallmarks of AD (Ising and Heneka, 2018). Aβ can further drive tau pathology by stimulating neuroinflammation and the neuroinflammatory response will aggravate the deposition of Aβ (Cai et al., 2014). *In vivo*, small and stable Aβ oligomers (AβOs) are thought to be more associated with the severity of AD’s neurodegeneration (Lue et al., 1999; McLean et al., 1999). With the increasing understanding of the inflammatory mechanisms in AD, the intervention of microglia activation and neuroinflammation provides a potential target for AD therapy. ABPPk is an active polypeptide fraction isolated from the herbal medicine *A. bidentate* with potential neuroprotective effects in ischemic stroke and Parkinson’s disease (Yu et al., 2014; Cheng et al., 2018; Peng et al., 2018). *In vitro* experiments revealed that ABPPk had an inhibitory effect on inflammatory response in LPS-induced BV2 microglia (Cheng et al., 2019). In the present study, we focused on the role of ABPPk in neuroinflammation and neurotoxicity induced by AβOs.

AβOs have an early role in AD pathology before the appearance of amyloid deposits (Klein et al., 2001; Ledo et al., 2016). A variety of receptors such as toll-like receptors expressed by microglia can bind AβOs to trigger neuroinflammation and produce inflammatory factors (Heneka et al., 2015; Heneka, 2017). BV2 microglia are one of the most commonly used immortalized cells with similar characteristics as primary microglia (Blasi et al., 1990). When stimulated by LPS or AβOs, BV2 microglia can be activated into different phenotypes and mediate inflammatory responses (Kopec and Carroll, 1998; Stansley et al., 2012; Park et al., 2013; Heindl et al., 2018). A single intracerebroventricular injection of AβOs to mouse has been widely used as an animal model to investigate the neurotoxicity of AβOs (Figueiredo et al., 2013; Ledo et al., 2013; Ali et al., 2015). In this study, we used AβOs-stimulated microglia and AβOs injected mouse model to investigate the effect of anti-neuroinflammation of ABPPk *in vitro* and *in vivo*, respectively.

Activation of microglia is accompanied by morphologic alternations from the ramified shape to amoeba-like shape (Lawson et al., 1990). We observed that 10 μM AβOs stimulation led to the morphologic changes of BV2 microglia with shorter and less branches, but AβOs-induced deformation was significantly reduced by the pretreatment of 10 μg/ml ABPPk. Activated microglia release a variety of soluble factors, including pro- and anti-inflammatory factors to regulate the neuroinflammation (Wes et al., 2016). We quantitatively analyzed the mRNA levels of inflammatory cytokines in the AβOs-stimulated BV2 microglia and found that 10 μM of AβOs significantly induced the up-regulation of pro-inflammatory IL-1β, IL-6, IL-18, and TNF-α, while ABPPk pretreatment inhibited the mRNA levels of them dose-dependently. As for the typical anti-inflammatory IL-4, IL-10, IL-13, and TGF-β, their mRNA levels were up-regulated with the pretreatment of ABPPk in a dose-dependent manner as compared to the AβOs-treated cells. The IC50 of ABPPk on AβOs-induced IL-1β release was about 8 μg/ml. Meanwhile, the inhibitory effect of ABPPk on AβOs-induced pro-inflammatory cytokines (TNF-α and IL-1β) release was also confirmed in primary cultured microglia.

Consistent with *in vitro* results, *in vivo* experiments showed that 2.5 mg/kg ABPPk significantly inhibited AβOs (80 μM) injection induced microglia activation in the hippocampal CA3 region of brain. AβOs injection resulted in a significant increase in the production of pro-inflammatory IL-1β and IL-18 in the brain, while ABPPk inhibited their production. AβOs induced a decrease in the production of anti-inflammatory IL-4, IL-10, and TGF-β, while ABPPk increased their production in the brain. These results suggested that pre-administration with ABPPk attenuates the inflammatory response induced by AβOs.

After intracerebroventricular injection, AβOs diffused largely in the brain as soon as 1 h and up to 7 days (Epelbaum et al., 2015). Toxic AβOs accumulate in the brain, leading to synapse failure, memory loss, and depressive-like behavior, which is closely related to the activation of inflammatory pathways (West et al., 1994; Ferreira and Klein, 2011; Viola and Klein, 2015; Ledo et al., 2016). The hippocampus is the main region damaged by AβOs (Brouillette et al., 2012). We found that ABPPk had no direct protective effect on hippocampal neurons insulted by AβOs *in vitro*, but stimulating hippocampal neurons with BV2 microglia CM system revealed that the neurotoxicity of 10 μg/ml ABPPk pretreated BV2 microglia CM decreased significantly compared with that from AβOs stimulated cells without ABPPk pretreatment. *In vivo*, we also demonstrated that ABPPk pre-administration significantly reduced the hippocampal neuronal degeneration caused by AβOs. Taken together, these results indicated that ABPPk protects against the neurotoxicity of AβOs on neurons through alleviating neuroinflammation.

AβOs link depressive-like behavior and cognitive deficits in mice (Ledo et al., 2013; Ledo et al., 2016). The open field test is a classical experiment performed to assess general locomotor, exploration, and anxiety-related behavior (Walsh and Cummins, 1976; Wu et al., 2020). The immobility time and the frequency of rearing are usually used to measure locomotor activity. The travelling distance and time spent in the central square are measured to determine exploratory behavior and anxiety. Our results showed that AβOs-injected mice exhibited a significant lower locomotor activity, lower exploratory behavior, and higher anxiety level compared with the sham group, while ABPPk pre-administrated mice had improved locomotor activity and less depressive-like mood. The Morris water maze is a widely accepted test of hippocampal-dependent learning, including acquisition of spatial memory and long-term spatial memory in animals (Bromley-Brits et al., 2011). In the probe trial, it was observed that the navigation ability of AβOs-injected mice was significantly reduced, indicating that they had a vague memory of the location of the target platform and its quadrant, while the mice in the ABPPk group showed improved behavior. Although there was no statistical difference between AβOs group and ABPPk group in the number of entering into the platform position, the ABPPk group still showed a trend of more times than AβOs group, which was probably due to the number of samples and the individual differences.

Inflammasomes are multiple protein complexes in the cytoplasm mainly containing pattern recognition receptors (PRRs), including NOD-like receptors (NLRs), Toll-like receptors (TLRs), C-type lectin receptors (CLRs), RIG-I like receptors (RLRs), and AIM2-like receptors (ALRs), apoptosis-associated speck-like protein containing a CARD (ASC), and pro-caspase 1. The assembly of inflammasome complexes begins with the NLRs or TLRs recognizing a specific stimulus (Kumar et al., 2011). Microglia express NLRP3 that can bind to Aβ and trigger neuroinflammation (Heneka et al., 2013). NLRP3 is unique among the NLRPs because its basal expression is not sufficient for activating inflammasomes in resting microglia, and it needs to be transcriptionally induced and activated to allow the assembly of NLRP3 inflammasomes. The key mediator of immunity, NF-κB, is necessary for the activation of NLRP3 (Bauernfeind et al., 2009). ASC is the platform for the activation of pro-caspase 1 into caspase 1, and the activated caspase 1 cleavages and processes the precursor of IL-1β and IL-18, making it mature and secreted extracellular (Martinon et al., 2002; Lamkanfi and Dixit, 2014). Transcription of pro-IL-1β is induced by the activation of NF-κB. The production of IL-1β is tightly dependent on NF-κB as well (Poeck et al., 2010). Therefore, inhibiting the activation of NF-κB is a key step to inhibit the formation of NLRP3 inflammasomes induced by Aβ. The NF-κB family has five subunits, and the phosphorylation of NF-κB p65 (Ser536) is required for the activation and nuclear translocation of NF-κB (Jin et al., 2019). We found that ABPPk inhibited the AβOs-induced increased levels of IL-1β and IL-18 both *in vitro* and *in vivo*. Consistent with previous studies, AβOs stimulation induced the phosphorylation and nuclear translocation of NF-κB p65 both *in vitro* and *in vivo*, but ABPPk suppressed the transcriptional activity of NF-κB p65. As expected, ABPPk inhibited NF-κB p65 activation, and NLRP3 expression was subsequently down-regulated.

Once activated, microglia present different polarization phenotypes including classic activated M1-phenotype and alternative activated M2-phenotype. M1 microglia can release harmful pro-inflammatory factors leading to neuronal degeneration and loss while M2 microglia can engulf and remove debris and release anti-inflammatory and neuroprotective factors to promote neuronal restoration after damage (Lawson et al., 1990). Emerging studies have shown that the polarization and function of microglia can be reversed in response to microenvironmental signals (Mosser and Edwards, 2008; Tanaka et al., 2020). Therefore, the regulation of the polarization of microglia has become the main target to treat neurodegenerative diseases such as AD (McGeer et al., 2016). LPS can promote M1-type polarization and restrict M2 phenotype activation (Ji et al., 2018). In this study, we observed the enhanced M1 marker-positive signal under the stimulation of LPS, whereas pretreatment of ABPPk inhibited the expression of M1 marker significantly. IL-4 can activate M2 phenotype polarization to accelerate the degradation and clearance of AβOs (Shimizu et al., 2008). We found that IL-4 induced the positive signal of M2 marker in BV2 microglia, and when ABPPk was present, the expression of M2 marker was significantly higher than that of cells treated with IL-4 alone. Increasing evidence suggests that microglial autophagy regulates neuroinflammation via modulating the phenotype shift of microglia (Ji et al., 2018; Ma et al., 2020; Zhang et al., 2020). The regulating effect of LPS on microglia polarization is related to its inhibition of microglia autophagy (Ji et al., 2018). Here, we demonstrated that LPS could inhibit the autophagy in BV2 microglia through up-regulating the expression of autophagosome marker SQSTM1/p62 in microglia and reducing the autophagic flux of LC3B. ABPPk pretreatment significantly inactivated SQSTM1/p62 and increased autophagic flux, which was similar to the classical autophagy activator rapamycin and activated LPS-inhibited autophagy in BV2 microglia. When autophagy was completely blocked by an autophagy inhibitor, 3-Methyladenine, the inhibition of M1-type polarization and the promotion of M2-type polarization by ABPPk on LPS-stimulated BV2 microglia were both eliminated.

Evidences from animal models and patients with AD suggest that long-term exposure to Aβ impairs microglial autophagy and the autophagy deficiency further affects the progression of AD disease (Lucin et al., 2013; Pomilio et al., 2020). In our study, 10 μM AβOs stimulation for 4 and 12 h activated the autophagy in BV2 microglia, while 10 μM AβOs stimulation for 24 h resulted in a decreased autophagy flux. However, pretreatment with 10 μg/ml ABPPk restored the AβOs-impaired autophagy in microglia. As expected, the regulatory effect of ABPPk on AβOs-stimulated microglia polarization disappeared when pretreated 3-MA completely blocked microglia autophagy. Taken together, the present results strongly suggested that autophagy restored by ABPPk is involved in the regulation of AβOs-stimulated microglia polarization.

There are some shortcomings in our study. The anti-inflammatory effect of ABPPk on AD model needs to be verified by other models, such as APP transgenic mice. Astrocytes also play an important role in neuroinflammation. Although previous studies have shown that ABPPk can reduce the activation of astrocytes in the PD model, the effect of ABPPk on astrocytic reactivity induced by Aβ oligomers stimulation remains to be studied.

## Conclusion

In the current study, we showed that ABPPk protects hippocampal neurons against AβOs-induced neurotoxicity through regulating microglial inflammation both *in vitro* and *in vivo*. We used polarization experiment to elucidate the underlying mechanism that ABPPk inhibited M1-polarization and promoted M2-polarization through enhancing the autophagy in microglia.

## Data Availability

The raw data supporting the conclusions of this article will be made available by the authors, without undue reservation.
