# Supplementary figures and images for: Anti-inflammatory Activity of a Polypeptide Fraction From Achyranthes bidentate in Amyloid β Oligomers Induced Model of Alzheimer’s Disease
Source: Front Pharmacol. 2021 Aug 12;12:716177. doi: 10.3389/fphar.2021.716177 (PMC8397449; doi:10.3389/fphar.2021.716177)

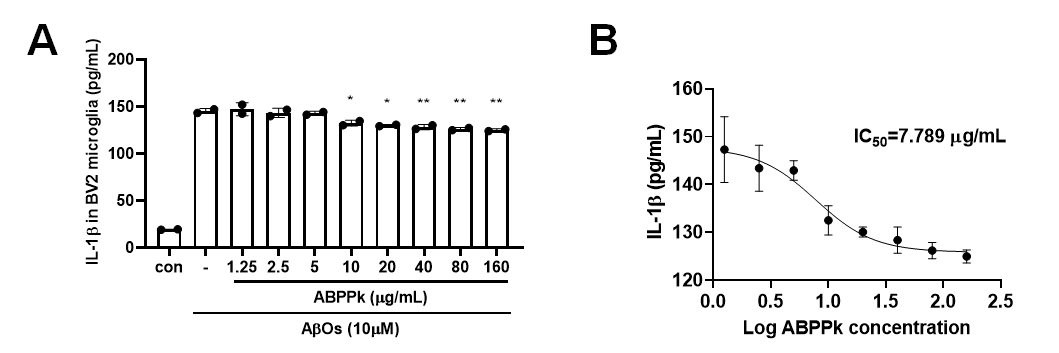

Supplement: Supplementary file 1 [file Image3.TIF]

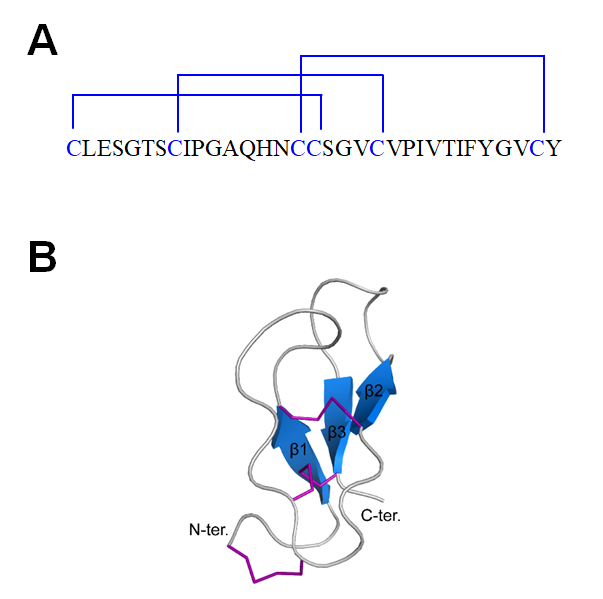

Supplement: Supplementary file 2 [file Image2.TIF]

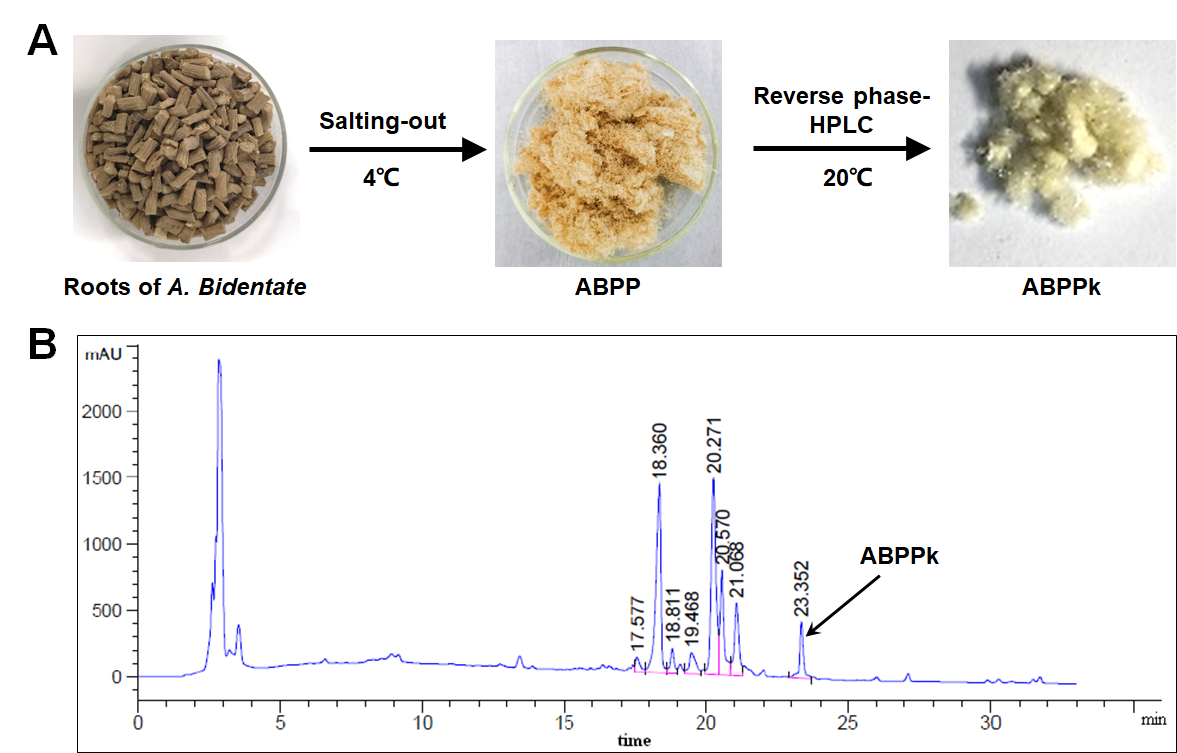

Supplement: Supplementary file 3 [file Image1.TIF]
